# Epigenetic aging markers in the association between frailty and mortality among U.S. adults

**DOI:** 10.1186/s12916-026-04866-0

**Published:** 2026-04-15

**Authors:** May A. Beydoun, Nicole Noren Hooten, Hind A. Beydoun, Michael F. Georgescu, Jack Tsai, Michele K. Evans, Alan B. Zonderman

**Affiliations:** 1 Laboratory of Epidemiology and Population Sciences, National Institute on Aging, NIA/NIH/IRP, Baltimore, MD 21224 USA; 2 VA National Center On Homelessness Among Veterans, U.S. Department of Veterans Affairs, Washington, DC 20420 USA; 3 Department of Management, Policy, and Community Health, School of Public Health, University of Texas Health Science Center at Houston, Houston, TX 77030 USA; 4 NIH Biomedical Research Center, National Institute On Aging Intramural Research Program, 251 Bayview Blvd, Suite 100, Baltimore, MD 21224 USA

**Keywords:** Frailty, Epigenetic clocks, Biological aging, Mortality, Additive Bayesian Networks

## Abstract

**Background:**

Frailty reflects diminished physiological reserve and increased vulnerability to adverse health outcomes. It has been linked to biological aging, including epigenetic age acceleration (EAA), a DNA methylation–based marker of aging, but the extent to which EAA accounts for the frailty–mortality association remains unclear.

**Methods:**

We analyzed three U.S. cohorts—NHANES (1999–2002), HRS (2016), and HANDLS (2004–2009)—with mortality follow-up through 2019–2022. Frailty was defined using harmonized adaptations of the Fried phenotype and FRAIL scale. EAA was derived from five epigenetic clocks (Horvath, Hannum, PhenoAge, GrimAge, DunedinPoAm). Additive Bayesian networks, Cox proportional hazards models, and counterfactual four-way decomposition were used to assess potential mediation and moderation of the frailty–mortality association by EAA, adjusting for age, sex, race/ethnicity, and socioeconomic status.

**Results:**

Frailty was strongly associated with higher all-cause mortality in NHANES and HRS. GrimAge and DunedinPoAm showed the strongest mediation. In NHANES, GrimAge accounted for 33% (p < 0.001) and DunedinPoAm mediated 17% (p = 0.006) of the association. In HRS, DunedinPoAm mediated 9% (p = 0.040) and GrimAge 16% (p = 0.020). Other clocks showed limited mediation. HANDLS findings were consistent. Higher socioeconomic status was associated with slower aging and lower frailty risk. Female sex was inversely associated with multiple epigenetic clocks but positively associated with frailty.

**Conclusions:**

Epigenetic aging, particularly GrimAge and DunedinPoAm, may explain part of the frailty–mortality association, supporting a role for biological aging pathways linking frailty to mortality.

**Supplementary Information:**

The online version contains supplementary material available at 10.1186/s12916-026-04866-0.

## Background

Frailty is a multidimensional clinical syndrome marked by decreased physiological reserve and increased vulnerability to adverse health outcomes [1]. Strongly associated with aging, frailty predicts a wide range of age-related conditions, including disability, hospitalization, and all-cause mortality [2]. Frailty is most commonly operationalized using physical and functional criteria—such as the Fried frailty phenotype (FFS), deficit accumulation frailty indices (FI), or related survey- and hospital-based adaptations, each capturing complementary aspects of vulnerability across aging populations[3]. Although frailty is traditionally measured using physical and functional indicators, accumulating evidence suggests that it also reflects underlying biological aging processes, including epigenetic regulation captured through DNA methylation patterns across the genome [4].

Over the past 7–8 years, several studies have directly linked frailty to DNA methylation–based markers of biological aging. Early epigenome-wide association studies (EWAS) demonstrated that frailty status is associated with differential methylation at CpG sites involved in inflammatory, metabolic, and stress-response pathways, providing molecular evidence that frailty reflects biological aging rather than functional decline alone [5]. Subsequent work has shown that frailty, whether measured using phenotype-based or index-based approaches—is associated with DNA methylation–derived epigenetic age acceleration (EAA), particularly for second- and third-generation clocks that capture multisystem physiological dysregulation [6, 7].

EAA quantifies the gap between an individual’s epigenetic age and their chronological age, with higher values indicating accelerated epigenetic aging [8, 9]. Elevated EAA has been associated with a broad spectrum of adverse outcomes, including cardiovascular disease, neurodegeneration, cognitive decline, and increased mortality risk [10–17]. Despite evidence that frailty and EAA may reflect overlapping aging pathways, only a handful of studies have directly examined whether EAA mediates the link between frailty and mortality [18].

To capture the complexity of these relationships, traditional regression models may be insufficient, as they often assume linearity and independence among predictors. Additive Bayesian networks (ABN), a probabilistic graphical modeling approach, offers a flexible, data-driven framework to uncover direct and indirect associations among variables without imposing strict parametric assumptions [19–22]. ABNs allow for the exploration of complex dependency structures, which is especially useful when investigating multifaceted pathways involving biological aging [19–22]. Furthermore, to test mediation and moderation simultaneously, four-way decomposition can determine the proportion of the total effect of an exposure such as frailty on the mortality risk outcome that is explained by pure mediation, pure interaction, both, or none (i.e. controlled direct effect) through or with EAA measures [23–25].

In this study, we use three large datasets─ two nationally representative samples of U.S. adults and a probability sample of urban adults residing in Baltimore city, MD─ all linked with death data to examine the interconnections among frailty, epigenetic age acceleration, and all-cause mortality. Specifically, we aim to: (1) investigate the association between frailty status and epigenetic aging markers; (2) assess whether EAA mediates or moderates the relationship between frailty and mortality; and (3) identify plausible biological pathways linking frailty, EAA, and mortality using additive Bayesian network modeling. By integrating causal discovery and epidemiological methods, this study advances our understanding of how physical frailty may exert long-term effects on mortality risk via epigenetic mechanisms, offering new mechanistic insights that can be used for future aging trajectory interventions.

## Methods

### Databases

#### National Health and Nutrition Surveys

The National Health and Nutrition Examination Survey (NHANES) is a series of cross-sectional, nationally representative surveys administered by the National Center for Health Statistics (NCHS), initiated in the early 1970 s (https://wwwn.cdc.gov/nchs/nhanes/default.aspx). In 1999, NHANES transitioned to a continuous, biennial data collection model [26]. Key anthropometric and physiological measurements were gathered through standardized physical examinations conducted within several mobile examination centers. This study utilized data collected between 1999 and 2002, with mortality follow-up obtained by linkage to the National Death Index through 2019. All NHANES data collection procedures during this period were conducted in accordance with rigorous ethical protocols, including informed consent, assurance of participant confidentiality, risk minimization, and approval by the NCHS Research Ethics Review Board.

#### Health and Retirement Study

The Health and Retirement Study (HRS) is a nationally representative, longitudinal panel study that investigates a wide range of factors influencing health, aging, and retirement among U.S. adults aged 50 and older (https://hrs.isr.umich.edu/about) [27]. Sponsored by the National Institute on Aging (NIA) and conducted by the University of Michigan, the HRS collects data biennially through structured interviews, with supplemental waves focusing on physical, biological, and psychosocial domains. The core dataset includes comprehensive self-reported and objectively measured variables obtained through the Enhanced Face-to-Face Interview (EFTF), as well as off-cycle components that capture biological markers of aging. For harmonized longitudinal analysis, the RAND HRS dataset provides a user-friendly, cleaned version of key variables across waves. In this study, we utilized data from the 2014 and 2016 waves, incorporating physical measures, psychosocial factors, and biomarkers, and linked them to mortality data from the National Death Index through 2019. All study protocols were approved by the University of Michigan Institutional Review Board and were conducted in accordance with strict ethical guidelines, including informed consent, confidentiality protections, and risk minimization procedures.

#### Healthy Aging in Neighborhoods of Diversity across the Life Span

The Healthy Aging in Neighborhoods of Diversity across the Life Span (HANDLS) study is a population-based, prospective cohort study designed to examine the influences of race and socioeconomic status (SES) on health disparities and aging trajectories in urban-dwelling adults. Initiated in 2004 by the National Institute on Aging Intramural Research Program (NIA/IRP), HANDLS recruited African American and White participants aged 30–64 years from neighborhoods in Baltimore, Maryland, using an area probability sampling design (https://handls.nih.gov/) [28]. The study collects longitudinal data including sociodemographic characteristics, clinical and cognitive assessments, psychosocial measures, dietary intake, and a broad range of biological markers. Physical measures and biospecimens (e.g., blood, saliva, and DNA) are obtained during structured clinical visits. For this analysis, we utilized data from baseline (Wave 1, 2004–2009) and follow-up waves through 2019, linked to mortality outcomes via the National Death Index (NDI). HANDLS study protocols received approval from the Institutional Review Board of the National Institutes of Health and were conducted in accordance with ethical standards, including informed consent, confidentiality protections, and risk minimization procedures. Appendix I in Additional File 1 provides more details on all three cohorts and Table 1 summarizes survey characteristics and measures used in each. In summary, NHANES and HRS are publicly available, de-identified datasets with prior IRB approval and participant consent; the present secondary analyses did not require additional IRB review. HANDLS was approved by the NIH Intramural Research Program IRB, and participants provided written informed consent.

**Table 1:** Cohort characteristics and measurements

Cohort	Study Design & Years	Age Range at Baseline	Frailty Measure	Epigenetic Aging Measures	Timing of DNAm & Frailty Assessment	Mortality Follow-up Source & Period	Final Analytic Sample Size
NHANES	Nationally representative, 1999–2002	≥ 50 years	Fried phenotype (adapted): BMI < 18.5, fatigue (DPQ), lifting difficulty, walking difficulty, low activity	Horvath, Hannum, PhenoAge, GrimAge (EAA residuals), DunedinPoAm	Baseline (1999–2002)	National Death Index, through 2019	1,537
HRS	Nationally representative, 2016 wave	≥ 50 years	Fried phenotype (adapted): BMI change equivalent to a weight loss of with weight loss > 10 lbs/year (2014–2016), grip strength, walking difficulty, CES-D exhaustion, low activity	Horvath, Hannum, PhenoAge, GrimAge (EAA residuals), DunedinPoAm	Frailty: Mainly 2016; DNAm: 2016	NDI + Exit interviews, through 2022	1,466
HANDLS	Urban probability sample, 2004–2009	30–64 years	FRAIL scale (fatigue, resistance, ambulation, illness, weight loss)	Horvath, Hannum, PhenoAge, GrimAge, DunedinPACE	Baseline (2004–2009)	National Death Index, through 2022	455

*Abbreviations*: *BMI* body mass index, *CES-D* Center for Epidemiologic Studies–Depression scale, *DNAm* DNA methylation, *DPQ* Depression Questionnaire, *EAA* epigenetic age acceleration, *FRAIL* Fatigue, Resistance, Ambulation, Illness, and Loss of weight scale, *HANDLS* Healthy Aging in Neighborhoods of Diversity across the Life Span, *HRS* Health and Retirement Study, *NDI* National Death Index, *NHANES* National Health and Nutrition Examination Survey

### Mortality linkage

All three cohorts—NHANES, HRS, and HANDLS—provide linkage to mortality outcomes via the NDI, enabling longitudinal analysis of vital status and time of death. In NHANES, mortality data are linked to participant records through NDI and provided in a mortality file that can be merged with demographic, health, and examination data for each survey wave. The HRS links mortality data using NDI through 2022, in addition to information from follow-up interviews and public records, with key death variables integrated into the Tracker file—this includes month and year of death, which can be merged with the Core survey and RAND longitudinal files. Similarly, the HANDLS study is linked to the NDI to ascertain mortality status and timing for participants through 2022. Therefore, death outcomes were derived from linkage to the National Death Index (NDI) for NHANES and HANDLS and from the National Death Index and HRS exit interviews for HRS, with follow-up through the specified censoring dates.

### Frailty score and status

Frailty was assessed across three U.S. cohort studies—HRS, NHANES, and HANDLS—using harmonized adaptations of established frailty phenotype frameworks. In the HRS, frailty was measured using the Fried phenotype model, which includes five criteria: unintentional weight loss, weakness, slowness, exhaustion, and low physical activity [1]. For the 2016 wave, unintentional weight loss ("shrinking") was operationalized using a calculated annualized change in body mass index (BMI) between 2014 and 2016, equivalent to a weight loss > 1.48 kg/m^−2^/year indicating the criterion. Weakness was assessed by average handgrip strength across four trials, with sex-specific cutoffs set at the 20th percentile (≤ 27.1 kg for men, ≤ 16.6 kg for women). Due to limited performance testing, slowness was approximated via self-reported difficulty walking several blocks. Exhaustion was based on a shorter version of the Center for Epidemiologic Studies-Depression (CES-D) questionnaire items, with endorsement of “everything was an effort” or “could not get going” indicating presence. Low physical activity was defined by self-reported frequency of vigorous and moderate exercise, with infrequent engagement (≤ 1–3 times/month or never) considered low. Each component was scored as present or absent (1/0), summed to a frailty score (0–5), and classified as robust (0), pre-frail (1–2), or frail (≥ 3).

In NHANES 1999–2002, which overlaps with epigenetic biomarker data, frailty was also defined using the Fried framework [1]. Because NHANES lacks longitudinal weight data, shrinking was proxied by BMI < 18.5 kg/m^2^. Exhaustion was identified using responses to DPQ items on fatigue and disinterest. Weakness was assessed via self-reported difficulty lifting 10 pounds, while slowness was defined by difficulty walking a quarter-mile. Low physical activity was based on responses indicating no or minimal walking/bicycling. As in HRS, a frailty score (0–5) and status (robust, pre-frail, frail) were calculated.

In HANDLS, frailty was defined using the five-item FRAIL scale: fatigue, resistance, ambulation, illness, and weight loss [29–31]. Fatigue and weight loss were derived from CES-D items (frequent fatigue and poor appetite, respectively). Resistance and ambulation were measured via self-reported difficulty climbing stairs and walking a quarter-mile respectfully. Illness was defined as the presence of five or more physician-diagnosed chronic conditions from a list of 11. One point was assigned per criterion present, yielding a score of 0–5. Participants were classified as robust (0), pre-frail (1–2), or frail (3–5). Those with ≥ 3 valid components were included in analyses. The ordinal version of the FRAIL score was used in part of the analysis. Appendix II of Additional File 1 details harmonized frailty algorithms as applied to NHANES, HRS and HANDLS studies. In most analyses, frail vs. pre-frail/robust was used as the main exposure of interest with part of the analyses including the frailty sum score ranging from 0 to 5.

### Epigenetic clocks and age acceleration measures

HRS, NHANES, and HANDLS have incorporated DNA methylation based epigenetic clocks. Employing the Illumina MethylationEPIC v1.0 BeadChip array for DNAm profiling. In HRS, five epigenetic clocks were computed: Horvath, Hannum, Levine (PhenoAge), GrimAge, and Dunedin Pace of Aging DNA methylation (DunedinPoAm). EAA was derived for the first four clocks by regressing DNAm age on chronological age and using the residuals, while DunedinPoAm, which inherently reflects aging pace, required no transformation. NHANES applied similar residual-based methods to calculate EAA for the same four clocks. HANDLS derived the same clocks mirroring those available in HRS and NHANES, with the exception of Dunedin Pace of Aging Calculated from the Epigenome (DunedinPACE). The computation of EAA via residuals was standardized across studies for consistency (see Appendix III of Additional File 1 for detailed methodology and potential adjustments for leukocyte cell counts as a sensitivity analysis). In NHANES and HANDLS, DNAm and frailty were measured contemporaneously, while in HRS, frailty (2016) was measured up to 4 years prior to DNAm collection (2016). A similar approach was adopted in an earlier study [32], and other studies used a similar set of clocks [14, 28, 33–35].

### Socio-economic status (SES) index

In both the HRS and NHANES datasets, educational attainment and household income were integrated into a composite socioeconomic status (SES) index using principal components analysis (PCA). The first principal component was extracted and standardized into a z-score for use in analyses. In the HRS, educational attainment was classified into five categories: “No degree,” “GED,” “High school graduate,” “Some college,” and “College degree or higher.” Household income and wealth were categorized into the following brackets: “ < $25,000,” “$25,000–$125,999,” “$125,000–$299,999,” “$300,000–$649,999,” and “ ≥ $650,000.” Total household wealth was derived from the RAND HRS dataset, which aggregates financial and non-financial assets (e.g., checking and savings accounts, bonds, IRAs, and property) and subtracts total debt to estimate net wealth. For the present study, household wealth values were taken from the 2016 HRS wave and measured at the household level.

In NHANES, educational attainment (assessed among adults aged 20 years and older) was grouped into: “Less than 9th grade,” “9th–11th grade,” “High school graduate or GED,” “Some college or Associate’s degree,” and “College graduate or above.” Household income was assessed using the poverty income ratio (PIR), a continuous variable representing the ratio of household income to the federal poverty threshold. PIR values below 1.0 indicate income below the poverty line, whereas values above 1.0 indicate income above poverty level. This continuous PIR measure was used as the household income indicator in our analysis.

In the HANDLS study, SES was summarized using two primary indicators: educational attainment and poverty status. Educational attainment was self-reported and categorized into: “Less than high school,” “High school or GED,” “Some college or vocational training,” and “College degree or higher.” Poverty status was determined at enrollment based on the 2004 U.S. federal poverty guideline [36] and operationalized as a binary variable indicating whether a participant’s household income fell below or at/above 125% of the federal poverty level. For the purposes of this study, educational attainment and poverty status were combined using principal components analysis (PCA) to derive a standardized composite SES index (z-score), consistent with approaches applied to HRS and NHANES data.

### Covariates

In this study, key demographic variables were treated as exogenous factors and included baseline self-reported age, sex (coded as 0 = Male, 1 = Female), and race/ethnicity. To enable cross-cohort comparability, race and ethnicity were harmonized across NHANES, HRS, and HANDLS wherever possible. Standardized categories included Non-Hispanic White (NHW), Non-Hispanic Black (NHB), and Hispanic, with an additional category for “Other” racial/ethnic groups. These categories were operationalized using three mutually exclusive dummy variables. In the HANDLS study specifically, race was self-identified and limited to NHW and NHB participants by design, enabling a focused investigation of various risk factors of health outcomes in an urban population. A sensitivity analysis adjusted for White Blood Cell (WBC) composition for NHANES and HRS four-way decomposition models, discussed later, focusing on largest available sub-classes of WBCs as percentage of total WBC. Due to heterogeneity in data available, in NHANES, this amounted to adjusting for percentages in lymphocytes, monocytes and neutrophils. In contrast, for HRS, adjustment was made for percentages in B cells, T cells, NK cells, monocytes and dendritic cells. Further details are provided in Appendix VII of Additional File 1, with Appendices IV through VI of Additional File 1 detailing statistical methodologies applied in this study.

### Study samples

Figure S1 (Additional File 2) presents participant flowcharts for the NHANES 1999–2002, HRS 2016, and HANDLS analytic samples. All three studies include epigenetic clock data for adults aged 50 and older. In HRS, the flowchart traces the progression from the initial RAND longitudinal dataset—comprising data from HRS and earlier cohorts dating back to 1992—to individuals aged 50 + in the 2016 wave, then to those with frailty data, and finally to participants with available epigenetic clock measures in the 2016 wave. In NHANES, participants aged 50 and older from the 1999–2002 cycles were selected, then narrowed to those with complete frailty and epigenetic data. In HANDLS, the sample selection began with the initial cohort recruited between 2004 and 2009, comprising urban-dwelling African American and White adults aged 30–64 at baseline. From this cohort, we selected participants who were aged 30-64y at Visit 1 (2004–2009) assessment, had completed frailty status measurements, and had available DNA methylation data for epigenetic clock calculation. Mortality follow-up in HANDLS extended up to 17 years. The final analytic samples included 1,537 participants in NHANES, 1,466 in HRS and 455 in HANDLS, all aged 50 + at baseline with complete data on frailty, epigenetic clocks, and relevant covariates, except for HANDLS for which baseline age ranged between 30-64y.

### Statistical methods

All statistical analyses were performed using Stata version 18.0 [37], with supplementary visualizations and advanced analytical methods conducted with R version 4.4.1 [38]. Data from three nationally representative or community-based cohorts—NHANES, HRS, and HANDLS—were harmonized to enable a stratified analyses focused on the role of frailty in all-cause mortality and its potential mediation (along with moderation) through DNA methylation (DNAm)-based epigenetic age acceleration (EAA) measures.

Initial analyses characterized the distribution of key variables, including frailty status [based on Fried criteria (NHANES and HRS) or FRAIL scale in HANDLS], DNAm-based EAA metrics (Horvath, Hannum, PhenoAge, GrimAge, DunedinPACE), mortality status, and covariates (age, sex, race/ethnicity, SES). Means, medians, standard deviations, interquartile ranges, and frequency distributions were computed. Histograms were examined to detect outliers, and standardized procedures were applied to remove them from continuous variables. Descriptive comparisons across cohorts used weighted linear regression for continuous variables and multinomial logistic regression for categorical variables, accounting for survey design features in HRS, NHANES, and HANDLS that are in common to all three cohorts, namely sampling weights. Pearson correlation coefficients were used to assess descriptive (non-inferential) relationships between EAA measures and the frailty sum score (0–5) across the three cohorts, with results visualized using correlation heatmaps. Analyses were unweighted due to methodological limitations in estimating weighted correlations.

Kaplan–Meier survival curves were generated for each cohort to estimate time to all-cause mortality, stratified by EAA measures and frailty status (0 = pre-frail/robust vs. 1 = frail). Sampling weights were applied to adjust for design complexities, and survival differences across strata were tested using Cox-regression based tests. These non-parametric methods allowed visual and statistical assessment of survival patterns across biological aging and frailty categories.

In our main analyses, we first fitted survey-weighted Cox regression models to evaluate the association between frailty status, EAA metrics, and SES z-score with all-cause mortality, adjusting for age, sex and race/ethnicity. Each of these listed predictors were entered separately. Proportional hazards assumptions were evaluated using Schoenfeld residuals. Models were stratified by study cohort and used attained age as the time scale. As a sensitivity analysis, Age-squared was entered into these models in addition to the main effect of age.

Second, to model the probabilistic interdependencies among frailty, EAA measures, and mortality risk, ABNs were constructed using a discrete-time survival framework. Data were transformed into person-period format with binary indicators for 2-year follow-up intervals. Variables were treated as Gaussian or binomial; continuous predictors were discretized using percentile-based binning, with median values representing each bin. The optimal network structure was determined by comparing model fit across configurations allowing one to three parent nodes per variable; the two-parent configuration was selected when improvement plateaued. This analysis was conducted on the HRS and NHANES cohorts and was not adjusted for sampling weights due to current ABN software limitations (Appendices IV and V of Additional File 1).

Third, we subsequently employed generalized structural equation modeling (GSEM) to replicate the ABN-derived networks and estimate standard errors for the identified paths. Additionally, parametric survival models using a Weibull distribution were fitted to assess the direct and indirect relationships among frailty, EAA measures, sociodemographic covariates, and mortality. These models adjusted for the complex sampling design (sampling weights, strata, and PSUs) and were compared to simpler models assuming a simple random sampling design (Appendix VI in Additional File 1).

Finally, to quantify the mediating role of each EAA measure in the frailty–mortality relationship, we implemented a four-way decomposition approach based on counterfactual mediation methods [23, 39]. This technique partitioned the total effect of frailty on mortality into: (1) the controlled direct effect, or CDE (2) the reference interaction, (3) the mediated interaction, and (4) the pure indirect effect through EAA. Identification of CDEs depends on assumptions of no unmeasured exposure–outcome, mediator–outcome, or exposure–mediator confounding, correct model specification, and consistency/positivity. Substantively, the CDE represents the effect of the exposure on the outcome when the mediator is fixed at a specified level for all individuals, namely at the mean of each EAA, isolating pathways not operating through the mediator [23, 39]. Analyses were adjusted for age, sex, race/ethnicity, and SES, and were carried out in for each cohort sample, using a Cox proportional hazards regression model as the final outcome equation (Appendix VII in Additional File 1). A Type I error rate of 0.05 was considered for statistical significance. Similar to ABN and GSEM, four-way decomposition was applied only to the NHANES and HRS cohort data.

Two main sensitivity analyses were carried out on four-way decomposition models. The first one considered binary frailty as the potential mediator or moderator in the epigenetic aging → mortality risk relationship, thus addressing bi-directionality of the relationships among frailty, epigenetic aging and mortality. The second sensitivity analysis was carried out on both frailty → epigenetic aging → mortality and epigenetic aging → frailty → mortality four-way decomposition models by including leukocyte cell count as an exogenous set of covariates. This approach distinguishes immune-inclusive from immune-independent epigenetic aging signals without removing biologically meaningful variation from the primary mediation models. Detailed methods and results are provided in Appendices VII and VIII within Additional File 1.

## Results

Table 2 summarizes baseline characteristics and mortality outcomes across NHANES (1999–2002; *n* = 1,537), HRS (2016; *n* = 1,415), and HANDLS (2004–2009; *n* = 455). NHANES and HRS participants were comparable with respect to mean age, sex distribution, racial/ethnic composition, and EAA metrics, expressed as residual-based measures ± SE. Frailty prevalence was modestly higher in NHANES (24.6%) than in HRS (22.7%), and mortality rates were correspondingly greater in NHANES (43.3 per 1,000 person-years; 95% CI: 39.8–47.0) than in HRS (24.7; 95% CI: 21.4–28.6). In contrast, HANDLS participants were substantially younger at baseline (mean age = 46.9 years), included a racially diverse urban population with a majority of non-Hispanic Black participants, and exhibited more favorable survival profiles. Consistent with this younger age structure, HANDLS showed a markedly lower frailty prevalence (7.3%) and a substantially lower mortality rate (10.2 per 1,000 person-years; 95% CI: 7.6–13.8), reflecting earlier life-course stages and reduced competing mortality risk relative to NHANES and HRS. SES z-scores and Dunedin pace of aging measures differed between cohorts and thus were not readily comparable.

**Table 2:** Study characteristics and mortality risk across three cohorts (NHANES, HRS and HANDLS)

	NHANES 1999–2002	HRS 2016	HANDLS 2004–2009
	Mean (SE)	Mean (SE) or %	Mean (SE) or %
**Demographics**	(n = 1,537)	(n = 1,415)	(n = 455)
Age	68.3 (0.3)	68.6 (0.4)	46.9 (0.8)***
Sex, % female	56.0	54.9	52.3
Race/ethnicity			
Non-Hispanic White	81.1	81.7	39.7^***^
Non-Hispanic Black	7.8	8.4	60.2
Hispanic	7.8	7.2	__
Other	3.2	2.7	__
**Epigenetic age acceleration metrics**	(n = 1,537)	(N = 1,415)	(n = 455)
HorvathAgeEAA	+ 0.32 (0.20)	+ 0.08 (0.20)	+ 0.18 (0.36)
HannumAgeEAA	−0.11 (0.18)	+ 0.19 (0.15)	−0.37 (0.37)
PhenoAgeEAA	−0.10 (0.26)	−0.24 (0.23)	__
GrimAgeEAA	−0.46 (0.19)	−0.47 (0.18)	__
DunedinPoAm^1^	+ 1.10 (0.005)	+ 1.07 (0.003)	+ 1.05 (0.011)
**SES z-score**^1^	+ 0.352 (0.055)	+ 0.217 (0.044)	+ 0.500 (0.067)
**Frailty status, %**^1^	24.6	22.7	7.3
	(N = 1,537)	(N = 1,413)	(N = 455)
Mortality rate, per 1,000 Person-years, with 95% CI	43.3 (39.8–47.0)	24.7 (21.4–28.6)	10.2 (7.6–13.8)^***^

*Abbreviations*: *CI* Confidence Interval, *DunedinPoAm* Dunedin Pace of Aging DNA methylation clock, *GrimAgeEAA* Grim DNA methylation Epigenetic Age Acceleration, *HANDLS* Healthy Aging in Neighborhoods of Diversity Across the Life Span, *HannumAgeEAA* Hannum DNA methylation Age, *HorvathAgeEAA* Horvath DNA methyalation Age Epigenetic Age Acceleration, *HRS* Health and Retirement Study, *n* unweighted sample, *NHANES* National Health and Nutrition Examination Surveys, *PhenoAgeEAA* Pheno DNA methylation Age Epigenetic Age Acceleration, *SE* Standard Error, *SES* Socio-economic status, based on educational attainment and income level

^1^In HANDLS, DunedinPACE was made readily available rather than DunedinPoAm. SES z-scores were not readily comparable across cohorts. Frailty was also measured differently and thus cannot be readily compared

^*^P < 0.05; ^**^P < 0.010; ^***^P < 0.001 for null hypothesis of no difference in means or proportions based on a set of bivariate linear or multinomial logit regression models with Cohort as the only predictor, comparing HRS to NHANES and HANDLS to NHANES while accounting only for sampling weights

Figure S2 (Additional file 3) and Supplementary Datasheet 1 (Additional file 4) show Pearson correlation matrices of standardized SES, frailty, and five epigenetic aging measures in NHANES and HRS. Frailty was positively correlated with PhenoAgeEAA, GrimAgeEAA, and DunedinPoAm in both cohorts, while SES was inversely associated with frailty and most aging metrics, especially in NHANES. GrimAgeEAA and DunedinPoAm showed the strongest inter-correlations and links to frailty, whereas HorvathEAA and HannumEAA were weakly related, particularly in HRS. Similar patterns were observed in HANDLS, where frailty was associated with DunedinPACE and lower SES, with minimal associations for HorvathEAA and HannumEAA.

**Fig. 1:**
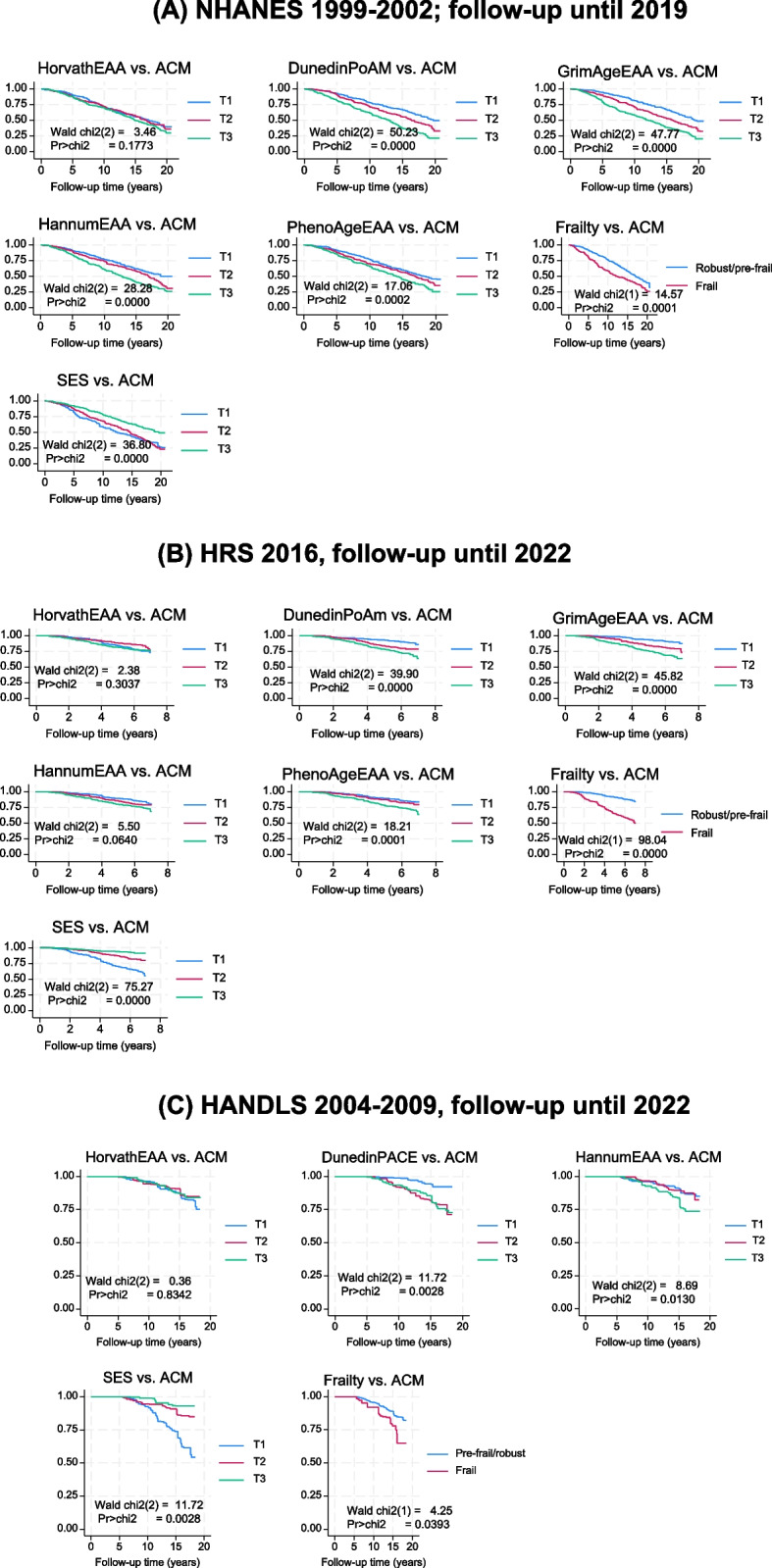
Kaplan–Meier survival curves across tertiles of SES, frailty, and markers of biological aging for the cohorts: NHANES 1999–2019, HRS 2016–2022 and HANDLS 2004–2022. *Abbreviations*: ACM = All-cause mortality; chi2 = Chi-square; DunedinPoAm = Dunedin Pace of Aging DNA methylation clock; GrimAgeEAA = Grim DNA methylation Epigenetic Age Acceleration; HANDLS = Healthy Aging in Neighborhoods of Diversity Across the Life Span; HannumAgeEAA = Hannum DNA methylation Age, Epigenetic Age Acceleration; HorvathAgeEAA = Horvath DNA methylation Age, Epigenetic Age Acceleration; HRS = Health and Retirement Study; NHANES = National Health and Nutrition Examination Surveys; PhenoAgeEAA = Pheno DNA methylation Age Epigenetic Age Acceleration; SES = Socio-economic Status; T1 = First tertile; T2 = Second Tertile; T3 = Third tertile. *Notes*: Kaplan–Meier survival curves were conducted in all three cohorts with time on study considered as the time variable to event (all-cause death) or censoring by end of follow-up. Maximum follow-up time ranged from ~ 8 years for HRS to 20 years for NHANES. Median values for tertiles (T1/T2/T3) were + 0.93 to 1.02/+ 1.065 to 1.10/+ 1.160 to + 1.21 for DunedinPoAm across 3 cohorts; −4.50 to −4.38/−0.86 to −0.75/+ 4.40 to + 4.56 for GrimAgeEAA across 2 cohorts; −5.38 to −4.58/−0.054 to + 0.381/+ 4.48 to 5.16 for HannumAgeEAA across 3 cohorts; −4.88 to −4.30/−0.108 to + 0.272/+ 4.40 to 5.45 for HorvathAgeEAA across 3 cohorts; −6.18 to −6.14/−0.294 to −0.008/+ 5.65 to + 6.33/for PhenoAgeEAA across 2 cohorts. SES z-scores have T1/T2/T3 corresponding approximately to a median of −1.12 to −0.94/−0.083 to + 0.38/+ 0.84 to + 1.20 for NHANES, HANDLS and HRS. Frailty status is included as a binary exposure (0 = pre-frail/robust vs. 1 = frail). Unweighted sample sizes were n = 1,537 for NHANES, n = 1,413 for HRS and n = 455 for HANDLS

Figure 1 displays Kaplan–Meier survival curves stratified by tertiles of SES, frailty status (binary), and epigenetic aging markers in NHANES (1999–2002; follow-up to 2019) and HRS (2016; follow-up to 2022). In NHANES, survival curves differed significantly by frailty status and all epigenetic clocks (HorvathAgeEAA, HannumAgeEAA, PhenoAgeEAA, GrimAgeEAA, and DunedinPoAm). SES showed an inverse relationship with all-cause mortality risk (p < 0.001). In HRS, similar patterns were observed: frailty and all five epigenetic markers were significantly associated with mortality (p < 0.001), while SES showed a strong inverse association with all-cause mortality (p < 0.001). These results suggest that higher frailty and accelerated biological aging—specifically as measured by DNA methylation clocks—are consistently associated with reduced survival across both cohorts, despite a longer follow-up observed in the NHANES (up to 20 years) compared to ~ 8 years in HRS. In HANDLS, SES z-score, frailty and DunedinPACE were among the strongest predictors for ACM, with no association detected for HorvathEAA.

**Fig. 2:**
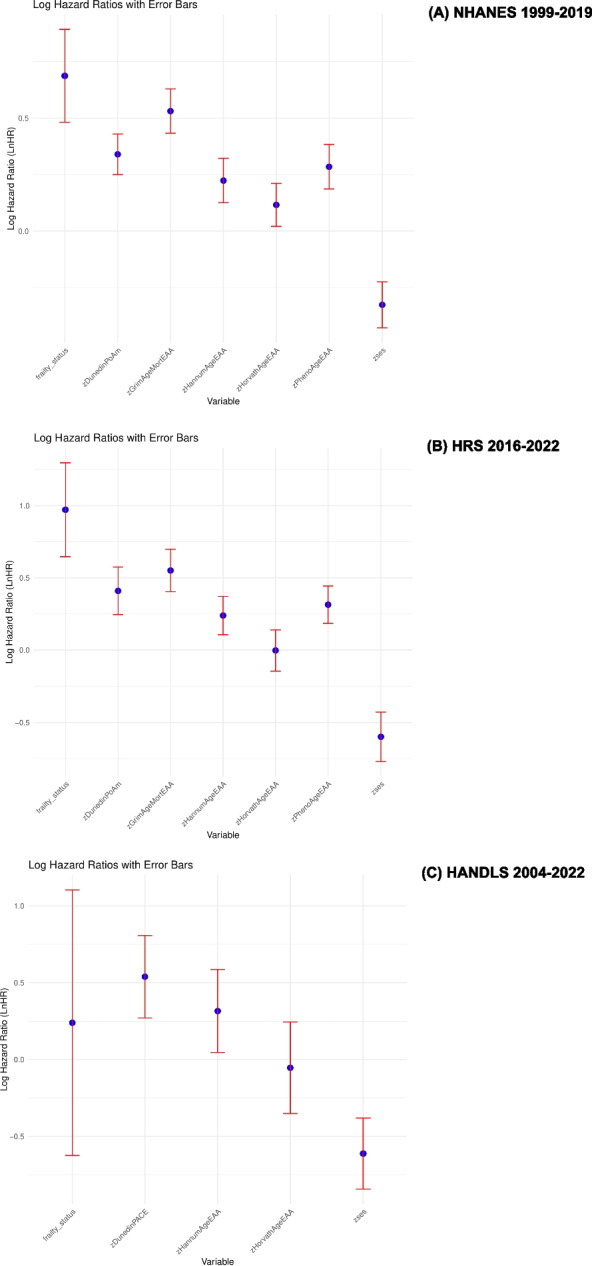
Association of each biological aging metric, frailty, and SES z-scores with mortality risk adjusting for key exogenous variables: Cox proportional hazards models. *Abbreviations*: DunedinPoAm = Dunedin Pace of Aging DNA methylation clock; GrimAgeEAA = Grim DNA methylation Epigenetic Age Acceleration; HANDLS = Healthy Aging in Neighborhoods of Diversity Across the Life Span HannumAgeEAA = Hannum DNA methylation Age, Epigenetic Age Acceleration; HorvathAgeEAA = Horvath DNA methylation Age, Epigenetic Age Acceleration; HRS = Health and Retirement Study; NHANES = National Health and Nutrition Examination Surveys; PhenoAgeEAA = Pheno DNA methylation Age Epigenetic Age Acceleration; SES = Socio-economic Status; z = standardized z-score. *Notes*: Models are adjusted for age, sex, and race/ethnicity within each cohort. Values are Ln(hazard ratios) with 95% CI for each biological aging metric. Sampling weights were accounted for in this analysis. Unweighted sample sizes were n = 1,537 for NHANES, n = 1,413 for HRS and n = 455 for HANDLS

Figure 2 and supplementary datasheet 2 (Additional File 5) present multivariable-adjusted Cox proportional hazards models examining associations between SES, frailty, and each of five epigenetic aging metrics, where available, with all-cause mortality in NHANES, HRS and HANDLS, adjusting for age, sex, and race/ethnicity. In NHANES, all epigenetic clocks were significantly associated with increased mortality risk. GrimAgeEAA showed the strongest association (HR = 1.70; 95% CI: 1.54–1.88), followed by DunedinPoAm (HR = 1.40; 95% CI: 1.28–1.54), PhenoAgeEAA (HR = 1.33; 95% CI: 1.20–1.47), HannumAgeEAA (HR = 1.25; 95% CI: 1.13–1.38), and HorvathAgeEAA (HR = 1.12; 95% CI: 1.02–1.23). In HRS, GrimAgeEAA (HR = 1.73; 95% CI: 1.50–2.01), DunedinPoAm (HR = 1.51; 95% CI: 1.28–1.78), PhenoAgeEAA (HR = 1.37; 95% CI: 1.20–1.56), and HannumAgeEAA (HR = 1.27; 95% CI: 1.11–1.45) were also significantly associated with elevated mortality risk, while HorvathAgeEAA was not (HR = 1.00; 95% CI: 0.87–1.15). Frailty status (i.e. frail vs. pre-frail/robust) was associated with nearly a twofold higher mortality risk in NHANES (HR = 1.99; 95% CI: 1.62–2.44) and over a two-and-a-half-fold risk in HRS (HR = 2.64; 95% CI: 1.91–3.66), independently of age, sex, and race/ethnicity. These findings underscore the robust predictive value of both frailty and advanced biological aging—especially as measured by GrimAge, PhenoAge, and DunedinPoAm—for mortality across nationally representative cohorts. In HANDLS, and unlike HRS or NHANES, frailty status was no longer associated with ACM upon adjustment for age, sex and race, in contrast with DunedinPACE, HannumAgeEAA, and SES. Inclusion of Age-squared into these models did not alter our main findings.

**Fig. 3:**
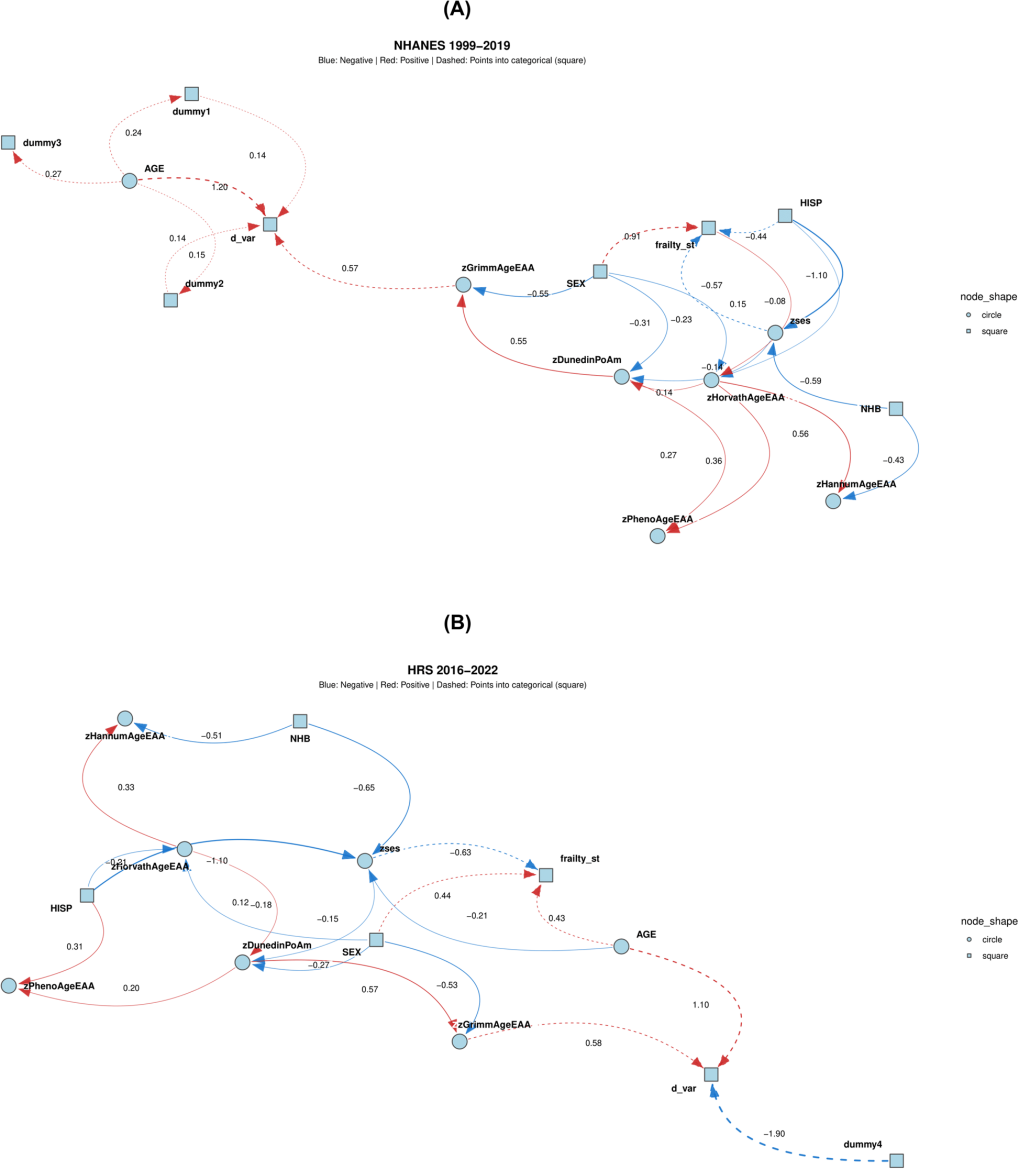
Additive Bayesian network solutions for 3 parents/child and model fit for 3 parents/child solution for associations among biological aging metrics, demographics, and mortality risk (discrete time hazards). *Abbreviations*: DunedinPoAm = Dunedin Pace of Aging DNA methylation clock; GrimAgeEAA = Grim DNA methylation Epigenetic Age Acceleration HannumAgeEAA = Hannum DNA methylation Age, Epigenetic Age Acceleration; HorvathAgeEAA = Horvath DNA methylation Age, Epigenetic Age Acceleration; HRS = Health and Retirement Study; NHANES = National Health and Nutrition Examination Surveys; PhenoAgeEAA = Pheno DNA methylation Age Epigenetic Age Acceleration; SES = Socio-economic Status; z = standardized z-score. *Notes*: This is a simplified version of the ABN solution. See Figure S3 for more details

Figure 3 and detailed Figure S3 (Additional File 6) compares ABN models from NHANES and HRS using the “three parents per child” specification, which allows up to three predictors for each non-exogenous variable. In both cohorts, model fit improved progressively from one to three parents per child, suggesting a more complex underlying network structure. Age and GrimAgeEAA consistently emerged as the strongest direct predictors of binary mortality risk. Female sex was associated with lower mortality, largely mediated through GrimAgeEAA or in combination with other epigenetic aging markers. SES was inversely related to biological aging as measured by DunedinPoAm in both cohorts. DunedinPoAm also showed a strong positive correlation with GrimAgeEAA, which directly predicted mortality. Notably, frailty status was positively associated with several EAA metrics and female sex, particularly in NHANES, reinforcing the relevance of biological aging as a correlate of frailty as a multidimensional clinical syndrome. While the configuration of interconnections among biological aging markers was moderately consistent across cohorts, simpler ABN models with only one or two parents per child (Figure S3: Additional File 6) produced sparser and less informative networks.

In both NHANES and HRS, GSEM derived from the 3-parent limit additive Bayesian network framework demonstrated consistent patterns of associations across biological aging, social determinants, and mortality (Table 3). Baseline age showed a robust and direct association with mortality risk in both cohorts (NHANES: β = 0.857, SE = 0.043; HRS: β = 1.099, SE = 0.092; both p < 0.001). GrimAgeEAA also significantly predicted mortality in NHANES (β = 0.541, SE = 0.054) and HRS (β = 0.522, SE = 0.068). Female sex was inversely associated with GrimAgeEAA, DunedinPoAm, and HorvathAgeEAA in both cohorts, indicating lower biological aging metrics in females, although it concurrently positively predicted frailty status (β = + 0.848, SE = 0.129 for NHANES, β = 0.418, SE = 0.133 for HRS). In both cohorts, higher SES predicted reduced frailty, even though frailty itself did not predict all-cause mortality in the ABN model and only marginally predicted HorvathAgeEAA in the NHANES prior to adjustment for sampling design complexity. Additionally, DunedinPoAm was strongly linked to other epigenetic clocks including GrimAgeEAA and PhenoAgeEAA, suggesting shared biological aging pathways. In both cohorts, SES predicted slower pace of aging as reflected by an inverse relationship with DunedinPoAm (e.g. β = −0.154, SE = 0.026 in HRS, without survey weighting). Across both samples, consistent directionalities and statistical significance were largely retained after adjusting for sampling design complexity.

**Table 3:** Generalized Structural Equations Models in NHANES, HRS and HANDLS samples based on the 3-parents/child limit Additive Bayesian Network Model solution for each cohort^a^

	**Model 1**^**b**^		**Model 2**^**c**^	
	β (SE)	P^d^	β (SE)	P^d^
**NHANES 1999–2019 (n = 1,537)**				
AGE → DIED	+ 0.831 (0.034)	< 0.001	+ 0.857 (0.043)	< 0.001
AGE → SES	−0.142 (0.023)	< 0.001	−0.134 (0.036)	< 0.001
SEX → Frailty	+ 0.848 (0.129)	< 0.001	+ 1.002 (0.208)	< 0.001
SEX → HorvathAgeEAA	−0.204 (0.052)	< 0.001	−0.187 (0.068)	< 0.001
SEX → DunedinPoAm	−0.291 (0.048)	< 0.001	−0.271 (0.058)	< 0.001
SEX → GrimAgeEAA	−0.525 (0.033)	< 0.001	−0.461 (0.039)	< 0.001
NHB → SES	−0.652 (0.061)	< 0.001	−0.668 (0.086)	< 0.001
NHB → HannumAgeEAA	−0.429 (0.046)	< 0.001	−0.317 (0.047)	< 0.001
HISP → Frailty	−0.542 (0.144)	< 0.001	−0.284 (0.260)	0.28
HISP → SES	−1.052 (0.052)	< 0.001	−1.063 (0.107)	< 0.001
HISP → HorvathAgeEAA	−0.092 (0.054)	0.085	−0.179 (0.067)	0.012
SES → Frailty	−0.573 (0.075)	< 0.001	−0.649 (0.104)	< 0.001
SES → DunedinPoAm	−0.130 (0.024)	< 0.001	−0.176 (0.029)	< 0.001
Frailty → HorvathAgeEAA	+ 0.114 (0.061)	0.064	+ 0.067 (0.077)	0.39
HorvathAgeEAA → DunedinPoAm	+ 0.129 (0.024)	< 0.001	+ 0.128 (0.038)	0.002
HorvathAgeEAA → HannumAgeEAA	+ 0.623 (0.019)	< 0.001	+ 0.598 (0.033)	< 0.001
HorvathAgeEAA → PhenoAgeEAA	+ 0.404 (0.022)	< 0.001	+ 0.415 (0.029)	< 0.001
HannumAgeEAA → PhenoAgeEAA	+ 0.301 (0.023)	< 0.001	+ 0.276 (0.043)	< 0.001
PhenoAgeEAA → GrimAgeEAA	+ 0.190 (0.018)	< 0.001	+ 0.214 (0.031)	< 0.001
GrimAgeEAA → DIED	+ 0.423 (0.033)	< 0.001	+ 0.541 (0.054)	< 0.001
DunedinPoAm → HannumAgeEAA	+ 0.131 (0.020)	< 0.001	+ 0.152 (0.024)	< 0.001
DunedinPoAm → GrimAgeEAA	+ 0.558 (0.018)	< 0.001	+ 0.567 (0.026)	< 0.001
DunedinPoAm → PhenoAgeEAA	+ 0.251 (0.018)	< 0.001	+ 0.253 (0.018)	< 0.001
**HRS 2016–2022 (n = 1,413)**				
AGE → DIED	+ 1.035 (0.067)	< 0.001	+ 1.099 (0.092)	< 0.001
AGE → SES	−0.211 (0.025)	< 0.001	−0.253 (0.033)	< 0.001
AGE → Frailty	+ 0.484 (0.064)	< 0.001	+ 0.561 (0.080)	< 0.001
.SEX → Frailty	+ 0.418 (0.133)	0.002	+ 0.339 (0.016)	< 0.001
SEX → HorvathAgeEAA	−0.181 (0.052)	< 0.001	−0.134 (0.073)	0.072
SEX → GrimAgeEAA	−0.515 (0.037)	< 0.001	−0.508 (0.048)	< 0.001
SEX → DunedinPoAM	−0.257 (0.052)	< 0.001	−0.278 (0.058)	< 0.001
NHB → SES	−0.631 (0.071)	< 0.001	−0.701 (0.107)	< 0.001
NHB → HannumAgeEAA	−0.486 (0.062)	< 0.001	−0.431 (0.087)	< 0.001
HISP → SES	−1.062 (0.082)	< 0.001	−1.144 (0.153)	< 0.001
HISP → HorvathAgeEAA	−0.191 (0.078)	0.016	−0.307 (0.047)	0.034
HISP → PhenoAgeEAA	+ 0.280 (0.075)	< 0.001	+ 0.327 (0.093)	0.001
SES → Frailty	−0.641 (0.070)	< 0.001	−0.729 (0.106)	< 0.001
SES → DunedinPoAm	−0.154 (0.026)	< 0.001	−0.180 (0.040)	< 0.001
HorvathAgeEAA → HannumAgeEAA	+ 0.351 (0.022)	< 0.001	+ 0.345 (0.041)	< 0.001
HorvathAgeEAA → DunedinPoAm	+ 0.114 (0.027)	< 0.001	+ 0106 (0.034)	0.003
HorvathAgeEAA → PhenoAgeEAA	+ 0.239 (0.024)	< 0.001	+ 0.220 (0.040)	< 0.001
PhenoAgeEAA → HannumAgeEAA	+ 0.340 (0.023)	< 0.001	+ 0.348 (0.039)	< 0.001
PhenoAgeEAA → GrimAgeEAA	+ 0.179 (0.019)	< 0.001	+ 0.180 (0.022)	< 0.001
GrimAgeEAA → DIED	+ 0.517 (0.057)	< 0.001	+ 0.522 (0.068)	< 0.001
DunedinPoAm → GrimAgeEAA	+ 0.578 (0.020)	< 0.001	+ 0.586 (0.027)	< 0.001
DunedinPoAm → PhenoAgeEAA	+ 0.210 (0.024)	< 0.001	+ 0.215 (0.028)	< 0.001

*Abbreviations*: *AGE* Baseline age, *DIED* Death event (yes vs. no), *DunedinPoAm* Dunedin Pace of Aging DNA methylation clock, *Frailty* Frailty status, *GrimAgeEAA* Grim DNA methylation Epigenetic Age Acceleration, *HannumAgeEAA* Hannum DNA methylation Age, Epigenetic Age Acceleration, *HISP* Hispanic, *HorvathAgeEAA* Horvath DNA methyalation Age, Epigenetic Age Acceleration, *HRS* Health and Retirement Study, *n* unweighted sample, *NHANES* National Health and Nutrition Examination Surveys, *NHB* Non-Hispanic Black, *HISP* Hispanic, *OTHER* Other race/ethnicities, *PhenoAgeEAA* Pheno DNA methylation Age Epigenetic Age Acceleration, *SE* Standard Error, *SEX* Female vs. Male

^a^Generalized structural equations models were conducted as a series of linear (most equations) and Weibull models (for the DIED outcome equation). The structure of each model was determined based on the 3-parent limit solution from ABNs for NHANES, HRS and HANDLS cohorts

^b^Model 1 was conducted without adjustment for sampling design complexity and thus assuming a simple random sample

^c^Model 2 adjusted for sampling design complexity by including sampling weights, PSU and strata that were most appropriate for each cohort

^d^P-value for null hypothesis that path coefficient β = 0. All p < 0.001 passed False Discovery Rate correction

**Fig. 4:**
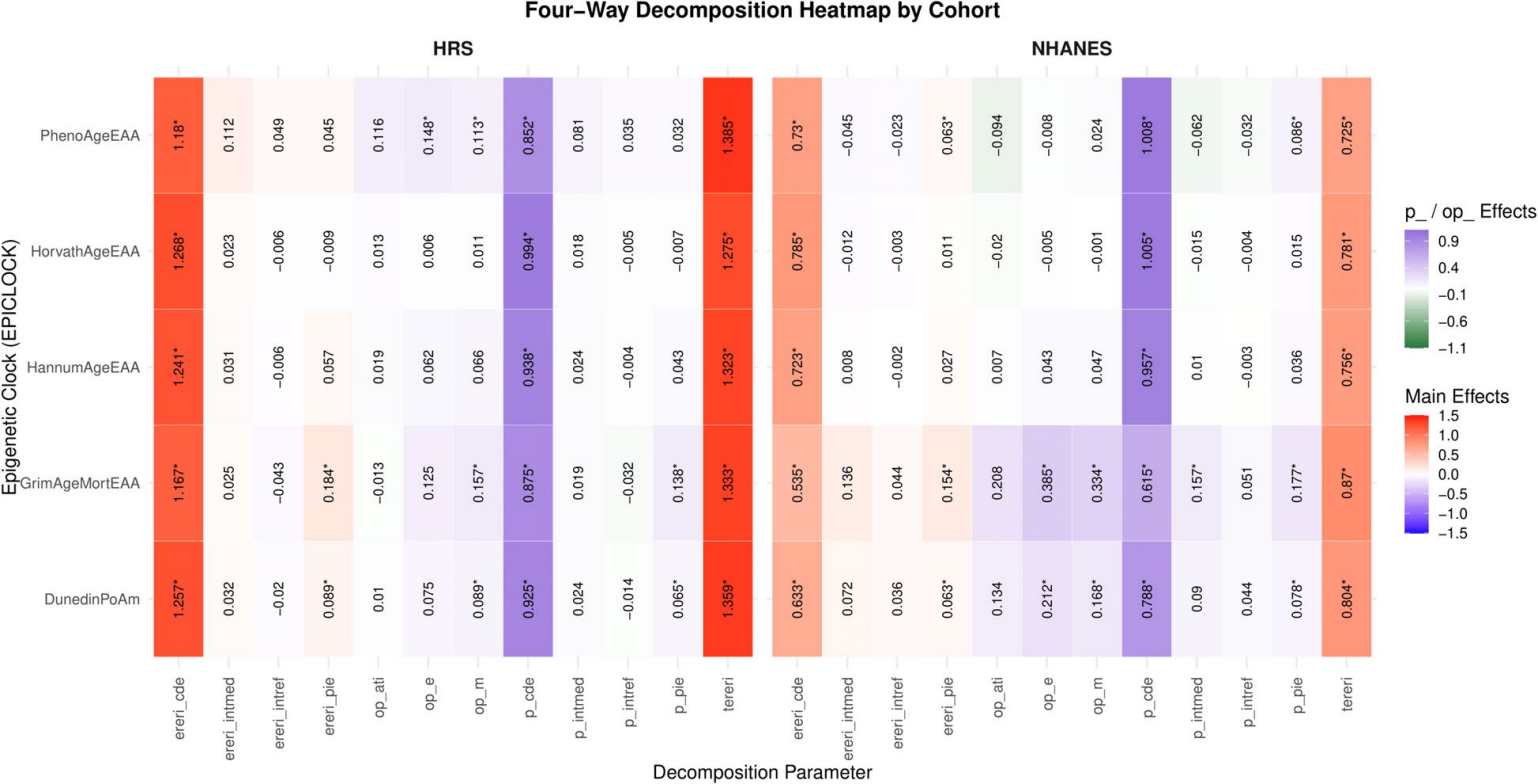
Heatmap of four-way decomposition models for frailty status and all-cause mortality by epigenetic age acceleration measures in NHANES (1999–2002, mortality follow-up through 2019) and HRS (2016, mortality follow-up through 2022). This figure displays standardized estimates from four-way decomposition models evaluating epigenetic age acceleration as a mediator of the association between frailty status and all-cause mortality. Rows represent epigenetic clocks and columns represent decomposition components. Color gradients reflect the magnitude and direction of effects (blue = negative; red = positive). Cell values denote point estimates, with asterisks indicating statistical significance (P < 0.05). Decomposition parameters include: *tereri* (total excess relative risk); *ereri_cde* (excess relative risk due to the controlled direct effect); *ereri_pie* (excess relative risk due to the pure indirect effect); *ereri_intmed* (excess relative risk due to mediated interaction); *ereri_intref* (excess relative risk due to reference interaction); *terira* (total effect risk ratio); proportion components (*p_cde, p_pie, p_intmed, p_intref*); and overall summary measures (*op_m*, overall proportion mediated; *op_ati*, overall proportion attributable to interaction; *op_e*, overall proportion eliminated). Models were adjusted for age, sex, race/ethnicity, and socioeconomic status index. Unweighted analytic sample sizes were n = 1,537 (NHANES) and n = 1,413 (HRS)

Through a series of multivariable-adjusted four-way decomposition models, and in both NHANES and HRS cohorts, frailty status was consistently associated with higher all-cause mortality risk, alternating five biological aging measures as potential mediators and/or moderators (Fig. 4, supplementary datasheet 3: Additional File 7). Total effect risk ratios ranged from 1.72 to 1.87 in NHANES and from 2.28 to 2.36 in HRS, indicating a robust relationship between frailty and mortality. Four-way decomposition revealed that the majority of the excess mortality risk was attributable to the controlled direct effect (CDE), accounting for over 90% of the total effect in models for 6 of 10 percentages controlled direct effects (P_CDE). Among the epigenetic clocks, GrimAgeEAA exhibited the strongest putatively mediating role in NHANES, with 33% of the association being potentially mediated (overall proportion mediated, op_m = 0.334, *p* < 0.001) under model assumptions and 38% of the effect eliminated under hypothetical intervention on the mediator (op_e = 0.385, *p* < 0.001). DunedinPoAm also demonstrated modest but statistically significant mediation effects in NHANES. In HRS, PhenoAgeEAA*,* DunedinPoAm and GrimAgeEAA emerged as modest mediators, with significant pure indirect effects and overall mediation (e.g., DunedinPoAm op_m = 0.089, *p* = 0.040). Conversely, HorvathEAA and HannumEAA clocks consistently showed weak or non-significant mediation effects in both cohorts. No meaningful contributions from reference or mediated interactions were detected. These findings suggest that specific epigenetic aging measures, particularly GrimAgeEAA and DunedinPoAm, may partially mediate the relationship between frailty and mortality, though the direct pathway remains predominant.

Reverse-causation models (epigenetic aging → frailty → mortality; Figure S4: Additional File 8) showed that second- and third-generation clocks had significant total associations with mortality, particularly GrimAge and DunedinPoAm. Frailty mediated approximately 8–18% of these associations across cohorts, with modest contributions from mediated interaction for advanced clocks. HorvathEAA showed little or no mediation. After adjustment for leukocyte composition (Figure S4: Additional File 8), total effects were modestly attenuated. In NHANES, mediation for GrimAge, DunedinPoAm, and PhenoAge remained largely intact. In HRS, attenuation was more pronounced, though GrimAge mediation persisted. Overall, findings indicate partial but consistent mediation by second- and third-generation clocks, with the direct frailty–mortality pathway remaining predominant. Detailed findings can be examined in supplementary datasheets 3 through 6 (Additional files 7, 9, 10 and 11), Appendix VIII of Additional File 1 and on Github.

## Discussion

### Summary of findings

In this study, we investigated the extent to which EAA may mediate or moderate the relationship between frailty and all-cause mortality using data from three large U.S. cohorts. Across NHANES, HRS, and HANDLS, frailty was consistently associated with a significantly elevated risk of mortality, independent of demographic and socioeconomic covariates. Among the five epigenetic clocks examined, GrimAge and DunedinPoAm emerged as the most salient mediators of this relationship. In NHANES, GrimAge explained approximately one-third (33%) of the total effect of frailty on mortality, while DunedinPoAm accounted for 17% of the association. In HRS, DunedinPoAm again demonstrated a modest but significant mediation effect, explaining 9% of the total effect. In contrast, Horvath and Hannum clocks showed minimal or non-significant mediation effects in both cohorts, suggesting that first-generation clocks may be less sensitive to biological changes linked to frailty and mortality. Notably, female sex was inversely associated with multiple epigenetic aging measures while being positively associated with frailty, highlighting a discordance between biological aging markers and clinical vulnerability. These findings support the hypothesis that accelerated biological aging, particularly as captured by GrimAge and DunedinPoAm, plays a partial but meaningful role in the pathway from frailty to mortality, while also suggesting that additional mechanisms beyond epigenetic aging contribute to mortality risk in frail individuals. These patterns were partly corroborated in HANDLS, despite the availability of only DunedinPACE among third-generation clocks. Overall, sensitivity analyses support the robustness of GrimAge, highlight heterogeneity across clocks, suggest partial bidirectionality, and reinforce cautious interpretation emphasizing shared biological aging processes rather than definitive mediation pathways.

### Previous studies

#### Frailty status and mortality

Numerous studies confirm that both baseline frailty and transitions toward frailty are associated with significantly higher mortality risk [40–42]. Importantly, frailty also modifies the effects of other risk factors on mortality, such as smoking [43], polypharmacy [44], and air pollution [45], suggesting a greater vulnerability to external and internal stressors. Longitudinal data highlight that improvements in frailty status may reduce mortality risk, whereas deterioration worsens outcomes [41, 42]. Findings across countries—including the U.S., Chile, China, and Europe—point to the global relevance of frailty as a strong mortality risk predictor [46, 47], with major implications for preventing premature death. In our study, frailty not emerging as a strong predictor in the ABN is likely due to shared variance that may have been absorbed by biological aging nodes, rather than being indicative of absence of association with all-cause mortality.

#### Association of socio-demographic and economic factors with frailty: epigenetic and other biological mechanisms

A substantial literature has established that socio-demographic and socioeconomic disadvantage is associated with frailty risk; more recent work has shifted toward understanding the biological pathways through which these effects are embedded. Lower educational attainment, income, and subjective social status are consistently linked to earlier frailty onset and progression [48, 49], largely through chronic psychosocial stress, adverse health behaviors, inflammation, and cardiometabolic dysregulation. Sex differences in these associations suggest that social support, caregiving roles, and cumulative stress exposures may differentially shape frailty trajectories in women and men [50, 51], while racial and ethnic disparities further reflect the impact of structural and cumulative disadvantage across the life course [52, 53]. Importantly, Mendelian randomization and cross-national studies strengthen causal inference by demonstrating that genetically proxied socioeconomic traits—particularly educational attainment—are associated with frailty burden [54, 55]. Emerging evidence indicates that epigenetic aging measures, especially clocks enriched for inflammatory and cardiometabolic pathways (e.g., GrimAge, DunedinPACE), may serve as key intermediates linking socioeconomic adversity to frailty by capturing the cumulative physiological toll of long-term disadvantage [35, 56–58]. Framing socioeconomic determinants within this biological aging context advances the field beyond descriptive associations toward identifying modifiable pathways for intervention.

Across the NHANES and HRS cohorts, higher socioeconomic status (SES) was consistently associated with a slower biological pace of aging and a lower likelihood of frailty, as evidenced by the inverse associations with DunedinPoAm and frailty risk. These findings align with a growing literature showing that socioeconomic advantage is linked to more favorable molecular aging profiles, likely through reduced chronic stress exposure, healthier behavioral patterns, and greater access to material and psychosocial resources that support physiological resilience. DunedinPoAm captures the coordinated rate of multisystem physiological decline and has been shown to be socially patterned, with faster aging observed among individuals exposed to socioeconomic disadvantage across the life course [35, 56–58]. In parallel, extensive epidemiologic evidence demonstrates that lower SES is a strong predictor of frailty, reflecting the cumulative biological wear and tear associated with long-term material hardship and psychosocial adversity [59, 60]. Together, these results support a model in which SES operates upstream of both molecular aging processes and clinical frailty, potentially linking social conditions to later-life vulnerability through accelerated biological aging pathways.

#### Association between frailty and epigenetic clocks

Emerging evidence indicates the role of epigenetic biomarkers in elucidating the biological underpinnings of frailty. Like our main findings, several studies have demonstrated that accelerated epigenetic aging, as measured by DNA methylation clocks, is significantly associated with higher frailty burden and worse clinical outcomes [61–63]. Among these biomarkers, GrimAge has shown superior predictive validity for frailty and mortality compared to other clocks [64, 65], a finding that resonates with our current study. Longitudinally, epigenetic age acceleration has also been linked to transitions in frailty over time [64, 66]. Mechanistic insights from systems biology suggest that frailty involves dysregulation of epigenetic pathways linked to inflammation, mitochondrial function, and oxidative stress[6, 67]. These findings and others provide a promising path toward individualized risk stratification and therapeutic strategies [68], highlighting the value of biological aging measures in geriatric care. More specifically, prior work has shown that CpGs contributing to GrimAge map strongly to genes involved in inflammatory and cardiometabolic processes, while DunedinPoAm/PACE captures coordinated methylation changes reflecting the pace of multisystem physiological decline [35, 69]. GrimAge and DunedinPoAm are derived from largely distinct CpG sets and were designed to capture overlapping but non-identical dimensions of biological aging. Their interconnections in the Bayesian network likely reflect shared upstream aging processes—such as chronic inflammation, immune senescence, and metabolic dysregulation—rather than direct causal effects of one clock on another. Given evidence of bi-directionality between epigenetic aging and frailty and ABN identifies conditional dependencies rather than definitive causal directions, our findings are consistent with both frameworks: (1) frailty contributing to accelerated molecular aging and (2) molecular aging dysregulation acting as an upstream risk factor for frailty [17, 70].

#### Cardio-metabolic risk, frailty and multimorbidity in relation to epigenetic clocks and mortality risk: Bi-directional relationships

A growing body of evidence supports epigenetic clocks as molecular intermediaries linking cardiometabolic risk, frailty, multimorbidity, and mortality. Studies of cardiovascular health—particularly those using the American Heart Association’s Life’s Essential 8 (LE8)—demonstrate that more favorable cardiovascular profiles are associated with lower epigenetic age acceleration (EAA), and that second-generation clocks such as PhenoAge and GrimAge partially mediate associations between cardiovascular health and both all-cause and cardiovascular mortality [71, 72]. Similarly, nonlinear associations between body mass index and survival appear to be partly explained by accelerated epigenetic aging, suggesting that DNAm-based aging metrics capture underlying metabolic dysregulation contributing to obesity-related mortality [71]. In life-course analyses, Klopack et al. showed that epigenetic aging measures mediate associations between cumulative smoking exposure and later-life chronic morbidity and mortality in the HRS [73]. Population-based studies further demonstrate that multiple epigenetic clocks mediate relationships between adverse lifestyle behaviors and premature death, reinforcing their role as integrative biomarkers of cumulative physiological stress [74]. In clinical and aging cohorts, greater multimorbidity burden and composite indices such as the VACS Index are associated with higher EAA, which in turn predicts mortality [18]. Collectively, these findings position epigenetic clocks as partial mediators translating cardiometabolic and multisystem risk into earlier mortality, although formal tests of bidirectional mediation between epigenetic aging and multisystem morbidity remain limited.

#### Integrated biological aging framework and translational implications for frailty–mortality pathways

Frailty can be conceptualized as a clinical expression of multisystem physiological dysregulation, whereas epigenetic clocks such as GrimAge and DunedinPoAm represent molecular summaries of cumulative biological stress. GrimAge incorporates CpG surrogates linked to inflammatory and mortality-related pathways, and DunedinPoAm reflects the coordinated pace of multisystem decline [35, 69]. Mortality, in turn, represents the downstream endpoint of accumulated vulnerability [75–77]. Rather than functioning as independent predictors, frailty, epigenetic aging, and mortality likely align along shared biological axes—phenotypic vulnerability, molecular aging signatures, and clinical outcomes—consistent with geroscience frameworks proposing common mechanisms across age-related conditions [75–77]. Mechanistically, chronic inflammation, immune senescence, oxidative stress, mitochondrial dysfunction, cardiometabolic dysregulation, and neuroendocrine stress signaling plausibly link these constructs [78, 79]. Although GrimAge and DunedinPoAm capture overlapping dimensions of these processes, they do not directly measure discrete biological pathways. In our four-way decomposition analyses, the controlled direct effect accounted for the majority (> 90% in many models) of the frailty–mortality association, with modest but significant mediation by GrimAge and DunedinPoAm and minimal interaction components. These findings suggest that epigenetic aging operates as a partial intermediary correlate rather than a dominant explanatory pathway. Stronger pathway-level inference will require longitudinal DNAm data, repeated frailty assessments, CpG-level mediation analyses, and integration with transcriptomic and proteomic profiling. Interventional studies targeting inflammatory burden or frailty trajectories will be essential to determine whether modifying biological aging processes meaningfully reduces mortality risk, thereby advancing translational relevance while maintaining appropriate causal caution.

### Strengths and limitations

This study offers several notable strengths. It investigates the association between biological aging and mortality risk in U.S. adults using three datasets. By integrating frailty, measured using standardized, harmonized instruments, alongside multiple EAA metrics, the study provides a comprehensive evaluation of how biological aging relates to mortality. The application of ABNs enhances the analysis by uncovering complex, probabilistic relationships among biological aging markers, sociodemographic characteristics, socioeconomic status, frailty, and mortality. The use of linked mortality data enables the investigation of long-term outcomes in relation to both clinical and molecular indicators of aging. Further methodological strength is added through the use of Cox regression models for survival analysis and GSEM to validate the inferred pathways.

Nonetheless, the present study has several limitations. These include cross-cohort heterogeneity in frailty and epigenetic measures (in definition, timing, and availability), reliance on primarily cross-sectional biomarker data, residual confounding due to incomplete harmonization of covariates (e.g., social relationship variables), and potential selection bias, including a healthy volunteer effect that may underestimate frailty prevalence and attenuate associations. Frailty and DNAm were measured contemporaneously (NHANES, HANDLS) or within a limited window (HRS). Although DNAm data theoretically allow derivation of multiple epigenetic biomarkers, the set of analyzable measures was constrained by cohort-specific factors, including differences in methylation arrays, CpG coverage, preprocessing pipelines, normalization procedures, and availability of calibration algorithms. For example, DunedinPACE was only available in HANDLS, whereas DunedinPoAm was available in NHANES and HRS. In addition, restricted access to raw DNAm data in NHANES and HRS, where only pre-computed epigenetic clock measures are available, limited the ability to derive alternative biomarkers. Consequently, we focused on clocks that were consistently available and harmonizable across cohorts to ensure comparability.

Furthermore, counterfactual mediation analyses rely on assumptions of no unmeasured confounding and correct temporal ordering; therefore, findings should be interpreted as consistent with intermediary processes rather than definitive causal pathways. Bidirectional relationships between frailty and epigenetic aging—supported by sensitivity analyses—further complicate causal interpretation. In this context, ABNs identify conditional dependencies but do not establish causal direction and should be viewed as hypothesis-generating.

Additional limitations include limited statistical power for cause-specific mortality and subgroup analyses, as well as the computational complexity of ABN modeling, which may affect replicability. The inability to include both frailty index (FI) and Fried frailty score (FFS) across all cohorts may have introduced additional heterogeneity; we prioritized FFS for consistency. Other differences in baseline age, clock availability, and follow-up duration may also affect cross-cohort comparability. Despite these limitations, this study provides robust, multi-cohort evidence supporting a role for epigenetic aging, particularly second- and third-generation clocks, as partial intermediaries in the frailty–mortality relationship.

## Conclusions

In summary, epigenetic aging, particularly as measured by GrimAge and DunedinPoAm, explained in part the relationship between frailty and all-cause mortality, identifying potential intermediary biological processes rather than definitive causal pathways. In NHANES, GrimAge explained about one-third of the effect, while DunedinPoAm showed smaller but significant potential mediating effects in both NHANES and HRS. These clocks outperformed first-generation measures (Horvath, Hannum), which showed minimal or undetectable associations, as corroborated by DunedinPACE in the HANDLS study. Notably, female sex was inversely associated with several epigenetic aging measures yet positively associated with frailty, suggesting a divergence between biological aging markers and clinical vulnerability. Despite partial mediation, most of the association was attributable to the direct effect, indicating that additional processes also contribute. These findings underscore the importance of addressing both frailty and epigenetic aging to reduce mortality risk in older adults. Future work leveraging CpG-specific pathway enrichment, transcriptomic integration, or locus-level mediation analyses may help elucidate the biological mechanisms underlying these associations and inform the development of frailty-sensitive or causally enriched epigenetic clocks.

## Supplementary Information

Additional file 1: Supplementary Appendices I–VIII. This file contains detailed methodological and supporting materials. Appendix I describes cohort design, sampling, and documentation for NHANES, HRS, and HANDLS. Appendix II details harmonized frailty definitions and algorithms across cohorts. Appendix III summarizes epigenetic clock derivation and age acceleration measures. Appendix IV outlines discrete-time hazard modeling. Appendix V describes the additive Bayesian network (ABN) framework and workflow. Appendix VI presents generalized structural equation modeling (GSEM) specifications. Appendix VII details four-way decomposition methods and assumptions, including sensitivity analyses with leukocyte adjustment. Appendix VIII summarizes sensitivity analysis findings across primary and reverse-causation models.

Additional file 2: Figures S1. Fig. S1 – Participant flowcharts for NHANES (1999–2002), HRS (2016), and HANDLS (2004–2009), showing sample selection and exclusions.

Additional file 3: Figure S2. Fig. S2 – Pearson correlation matrices and kernel density plots of frailty scores across NHANES, HRS, and HANDLS, illustrating relationships among SES, frailty, and epigenetic aging measures.

Additional file 4: Supplementary Datasheet 1. Supplementary Datasheet 1 – Correlation matrices and descriptive statistics for SES, frailty, and epigenetic aging measures across cohorts.

Additional file 5: Supplementary Datasheet 2. Supplementary Datasheet 2 – Multivariable Cox regression results for associations between SES, frailty, epigenetic clocks, and all-cause mortality across NHANES, HRS, and HANDLS.

Additional file 6: Figure S3. Fig. S3 – Additive Bayesian network (ABN) models for NHANES and HRS under varying parent limits (1–3 parents per node), including final network structures and model fit comparisons across specifications.

Additional file 7: Supplementary Datasheet 3. Supplementary Datasheet 3 – Detailed four-way decomposition results (primary models) for NHANES and HRS, including total effects and component estimates.

Additional file 8: Figure S4. Fig. S4 – Heatmaps summarizing four-way decomposition results for frailty–epigenetic aging–mortality pathways across primary and sensitivity analyses, including leukocyte-adjusted and reverse-causation models.

Additional file 9: Supplementary Datasheet 4. Supplementary Datasheet 4 – Four-way decomposition results with leukocyte (WBC) adjustment across NHANES and HRS.

Additional file 10: Supplementary Datasheet 5. Supplementary Datasheet 5 – Reverse-causation four-way decomposition results (epigenetic aging → frailty → mortality).

Additional file 11: Supplementary Datasheet 6. Supplementary Datasheet 6 – Reverse-causation four-way decomposition results with additional leukocyte (WBC) adjustment.

## Data Availability

NHANES and HRS data are publicly available at: [https://www.cdc.gov/nchs/nhanes/index.htm] and [https://hrs.isr.umich.edu/about], respectively. HANDLS data are available upon reasonable request through a manuscript proposal process ([https://handls.nih.gov/]). Code and analytic outputs supporting the findings of this study are available from the corresponding author upon reasonable request and will be made publicly available on GitHub at: [https://github.com/baydounm/HRS/_NHANES/_HANDLS/_FRAILEPIGENMORT].

## Abbreviations

ABN: Additive Bayesian Networks
ACM: All-cause mortality
BMI: Body mass index
CDE: Controlled direct effect
CES-D: Center for Epidemiologic Studies Depression Scale
CI: Confidence interval
CpG: Cytosine–phosphate–guanine site
DAG: Directed acyclic graphs
DIED: Death event (binary outcome)
DNAm: DNA methylation
DNAm age: DNA methylation age
DPQ: Depression Questionnaire
DunedinPACE: Dunedin Pace of Aging Calculated from the Epigenome
DunedinPoAm: Dunedin Pace of Aging DNA methylation
EAA: Epigenetic age acceleration
EFTF: Enhanced Face-to-Face Interview
EWAS: Epigenome-wide association study
FFS: Fried frailty score / Fried frailty phenotype
FI: Frailty index
FRAIL: Fatigue, Resistance, Ambulation, Illness, Loss of weight scale
GrimAgeEAA: Grim DNA methylation age epigenetic age acceleration
GSEM: Generalized structural equation modeling
GWAS: Genome-wide association studies
HANDLS: Healthy Aging in Neighborhoods of Diversity across the Life Span
HannumAgeEAA: Hannum DNA methylation age epigenetic age acceleration
HISP: Hispanic
HorvathAgeEAA: Horvath DNA methylation age epigenetic age acceleration
HR: Hazard ratio
HRS: Health and Retirement Study
INTmed: Mediated interaction
INTref: Reference interaction
IQR: Interquartile range
IRB: Institutional Review Board
Kaplan–Meier: Non-parametric survival estimator
LASSO: Least Absolute Shrinkage and Selection Operator
LE8: Life’s Essential 8
Ln: Natural logarithm
MRV: Medical Research Vehicles
NCHS: National Center for Health Statistics
NDI: National Death Index
NDE: Natural direct effect
NHB: Non-Hispanic Black
NHANES: National Health and Nutrition Examination Survey
NHW: Non-Hispanic White
NIA: National Institute on Aging
NIE: Natural indirect effect
NIH: National Institutes of Health
op_e: Proportion eliminated
op_m: Overall proportion mediated
OTHER: Other race/ethnicities
PCA: Principal components analysis
P_CDE: Percentage controlled direct effect
PhenoAgeEAA: Pheno/Levine DNA methylation age epigenetic age acceleration
PIE: Pure indirect effect
PIR: Poverty income ratio
Person-years: Time-at-risk metric in survival analysis
PSU: Primary sampling units
qPCR: Quantitative polymerase chain reaction
RAND: Research and Development (RAND HRS dataset)
SD: Standard deviation
SE: Standard error
SES: Socioeconomic status
TE: Total effect
Tracker file: HRS mortality linkage dataset
U.S.: United States
VA: Department of Veterans Affairs
WBC: White blood cell
Weibull model: Parametric survival model
Z-score: Standardized score
