# Supplementary material for: Epigenetic aging markers in the association between frailty and mortality among U.S. adults
Source: BMC Med. 2026 Apr 15;24:323. doi: 10.1186/s12916-026-04866-0 (PMC13192009; doi:10.1186/s12916-026-04866-0)
Supplement: Supplementary file 1 — Additional file 1: Supplementary Appendices I–VIII. This file contains detailed methodological and supporting materials. Appendix I describes cohort design, sampling, and documentation for NHANES, HRS, and HANDLS. Appendix II details harmonized frailty definitions and algorithms across cohorts. Appendix III summarizes epigenetic clock derivation and age acceleration measures. Appendix IV outlines discrete-time hazard modeling. Appendix V describes the additive Bayesian network (ABN) framework and workflow. Appendix VI presents generalized structural equation modeling (GSEM) specifications. Appendix VII details four-way decomposition methods and assumptions, including sensitivity analyses with leukocyte adjustment. Appendix VIII summarizes sensitivity analysis findings across primary and reverse-causation models. [file 12916_2026_4866_MOESM1_ESM.pdf]

# SUPPLEMENTARY MATERIALS

## APPENDIX I. DATABASES AND DETAILED STUDY DESIGN DOCUMENTATION

### 1) NHANES:

The CDC's NHANES website provides comprehensive resources to support data interpretation, including documentation on survey methodology, sample design, estimation procedures, and analytical strategies. These materials are regularly updated to reflect changes in survey structure and advances in statistical methods. Key components of NHANES analytic resources include the Plan and Operations Reports, Sample Design Documentation, Estimation and Weighting Procedures, and overarching Analytic Standards. The Analytic Guidelines for 1999–2010 offer detailed instructions for analyzing data from that period, while the Analytic Guidance for the 2017–March 2020 Pre-pandemic Data Files addresses challenges encountered during the COVID-19 pandemic and provides guidance for combining multiple survey cycles to produce nationally representative estimates.
For the present study, we used demographic data from the 1999–2000 and 2001–2002 cycles, which were merged with additional serum biomarker data to generate epigenetic clock measures. Although the demographic files cover individuals aged 0 to 85+, our analysis was restricted to participants aged 50 years and older, consistent with the availability of DNA methylation (DNAm) data. Most analyses employed a two-cycle weighting scheme, using four-year sample weights adjusted to account for the subset of participants with available epigenetic measures.

*Source*: https://wwwn.cdc.gov/nchs/nhanes/analyticguidelines.aspx

### 2) HRS:

The Health and Retirement Study (HRS) offers extensive documentation on its survey design and methodology. The Data Collection Path Table provides a historical overview of HRS data initiatives, with links to detailed information on each dataset. The longitudinal cohort sample design illustrates how the HRS integrates successive birth cohorts over time, maintaining a steady-state sampling approach by refreshing the study population with younger cohorts every six years. Detailed tables summarize sample sizes and interview response rates by survey wave, panel, race/ethnicity, and cohort. Survey weights are provided to ensure nationally representative estimates.
To support data management and analysis, resources such as An Elementary Cookbook of Data Management using HRS Data with SPSS, SAS, and Stata Examples offer practical guidance across statistical software. Technical reports elaborate on survey design, including the use of unfolding brackets to reduce item nonresponse and the imputation methods used across waves. Administrative materials cover IRB protocols and other governance-related considerations.
For this project, we used the 2020 HRS RAND FAT FILE, V1.A, released in May 2024, and the Cross-Wave Tracker File, updated in November 2024, which includes data through early 2022. The Tracker File provides a unique record per respondent and is used here to determine death dates and follow-up time for survival analyses. RAND and Tracker File resources are accessible at:

- RAND HRS: https://hrsdata.isr.umich.edu/data-products/rand
- Tracker File: https://hrsdata.isr.umich.edu/data-products/cross-wave-tracker-file

*Source*: https://hrs.isr.umich.edu/documentation/survey-design

**3) HANDLS**

The Healthy Aging in Neighborhoods of Diversity across the Life Span (HANDLS) study is a population-based, prospective cohort launched by the National Institute on Aging Intramural Research Program (NIA/IRP) in 2004. Its primary goal is to investigate the interplay between race, socioeconomic status (SES), and health disparities across the life course, particularly in urban environments. The HANDLS cohort comprises 3,720 community-dwelling adults aged 30–64 years at baseline, recruited from 13 neighborhoods in Baltimore, Maryland. Participants were selected using an area probability sampling strategy across a factorial design of sex, race (Black and White), age group, and poverty status (above or below 125% of the federal poverty level), ensuring diverse representation.

*Source*: https://handls.nih.gov/

**APPENDIX II. FRAILTY ALGORITHM AND DOCUMENTATION**

Frailty was initially operationalized using the Fried frailty phenotype, a widely used clinical model that characterizes frailty as a biological syndrome resulting from cumulative declines across multiple physiological systems. This model defines frailty based on the presence of three or more of the following five criteria, originally proposed by Fried et al. (2001) (1):

1. Unintentional weight loss (shrinking): Self-reported unintentional weight loss of >10 pounds (4.5 kg) in the past year, or a significant decline in body mass index (BMI), operationalized here as an annualized BMI decrease of $\geq$1.5 kg/m$^2$.
2. Weakness: Assessed via handgrip strength, defined as being in the lowest 20th percentile, adjusted for sex and body mass index.
3. Exhaustion: Based on self-reported items from the Center for Epidemiologic Studies Depression (CES-D) scale, indicating a frequent feeling of effort or inability to get going.
4. Slowness: Measured via gait speed or walking time over a specified distance (e.g., 2.5 or 4 meters), with the slowest 20% adjusted for sex and height considered frail.
5. Low physical activity: Determined using questionnaire data on weekly energy expenditure or frequency of engagement in moderate or vigorous activities, with thresholds based on sex-specific cut-points.

Both NHANES and HRS used a modified version of the Fried frailty phenotype. In HANDLS, another definition was used as described in a later section.

*Other source*: https://my.clevelandclinic.org/health/diseases/frailty

**1) NHANES**

We operationalized the Fried frailty phenotype based on five domains—shrinking, weakness, slowness, low physical activity, and fatigue—using available proxy variables from NHANES 1999–2000 and 2001–2002. The analysis combined relevant datasets across cycles, generated indicators for each frailty component, and constructed a summary frailty score and categorical frailty status. Below is a summary of the procedure:

Data Preparation and Merging

1. **Import and Append Data Files:**
    - The following NHANES data files were extracted and appended across cycles: *BMX (Body Measures)*, *PAQ (Physical Activity)*, *PFQ (Physical Functioning)*, and *DEMO (Demographics)*.
    - Each dataset was appended across cycles 1 (1999–2000) and 2 (2001–2002) after tagging them with a cycle variable.
    - The final working dataset was generated by sequentially merging DEMO with BMX, PAQ, and PFQ using SEQN as the key.

**Frailty Component Definitions**

Each frailty component was operationalized using available NHANES variables as follows:

1. Shrinking
   *Proxy:* Body Mass Index (BMI)
   *Variable:* BMXBMI
   *Definition:* Participants with BMI < 18.5 were coded as frail for shrinking.
2. Weakness
   *Proxy:* Self-reported difficulty lifting or carrying 10 pounds
   *Variable:* PFQ060E
   *Definition:* Responses of "some difficulty", "much difficulty", or "unable to do" were coded as frail.
3. Slowness
   *Proxy:* Difficulty walking a quarter mile
   *Variable:* PFQ060B
   *Definition:* Same response scheme as weakness; difficulty of any kind coded as frail.
4. Low Physical Activity
   *Proxies:* Engagement in vigorous or moderate physical activity in the past 30 days
   *Variables:* PAD200 (vigorous), PAD320 (moderate)
   *Definition:* Respondents answering "no" or "unable to do" for both items were classified as having low activity.
5. Fatigue
   *Proxy:* Difficulty walking up ten steps without resting
   *Variable:* PFQ060C
   *Definition:* Any reported difficulty (some, much, or unable) coded as frail.

**Score Construction**

- Each domain was scored as 1 (frail) or 0 (not frail).
- A frailty score was computed by summing the five components (range: 0–5).
- A frailty status categorical variable was then generated:
    - 0 = Robust (score = 0)
    - 0 = Pre-frail (score = 1–2)
    - 1 = Frail (score ≥ 3)

**Final Output**

The resulting dataset FRAILTY_NHANES.dta includes:

- Component variables (e.g., frail_weakness, frail_activity)
- The continuous frailty score (frailty_score)
- Categorical frailty status (frailty_status)

All analyses were conducted in Stata using syntax for data import (import sasxport5), appending, merging, recoding, and labeling consistent with NHANES documentation.

**2) HRS**

We constructed the Fried frailty score using data from the 2016 wave of the Health and Retirement Study (HRS), combining the RAND longitudinal file with the 2016 core physical measures (H16I_R) and psychosocial (H16LB_R) datasets. Data were preprocessed and merged using the unique household-person identifier (HHIDPN).

Fried frailty components were operationalized as follows:

1. Shrinking (Unintentional Weight Loss or being underweight): Calculated from BMI change between 2014 and 2016, with weight loss >10 lbs/year coded as 1 or being underweight in 2016 with BMI<18.5 kg.m$^{-2}$.
2. Weakness: Average grip strength across four trials (PI816, PI851–853); weakness was defined as being in the lowest sex-specific quintile (≤27.1 kg for men, ≤16.6 kg for women).
3. Slowness: Proxy based on self-reported difficulty walking several blocks (r13walksa).
4. Exhaustion: Derived from two CES-D items: "felt everything was an effort" (PLB018G) and "could not get going" (PLB018J); endorsement of either item (agree/strongly agree) classified as exhaustion.
5. Low Physical Activity: Defined as low frequency of vigorous or moderate physical activity (≤3 times per month or never, based on r13vgactx and r13mdactx).

Each component was coded as 1 (present) or 0 (absent), and summed to create the Fried frailty score (range 0–5). Based on established thresholds:

- 0 = Robust
- 1–2 = Pre-frail
- 3–5 = Frail

The final analytic dataset (hrs2016_friedfrailty_final.dta) includes individual component variables, total frailty score, and frailty status label. This approach aligns with prior research applying Fried's criteria in aging cohorts and facilitates integration with other HRS biomarker and epigenetic data.

**3) HANDLS**

The FRAIL scale comprises five domains: fatigue, resistance (ability to climb stairs), ambulation (ability to walk a specific distance), number of illnesses, and weight loss. We followed the original operationalization described by Morley et al., with the exception of the weight loss domain, which was adapted according to Theou et al.. Fatigue was assessed using item 20 from the Center for Epidemiologic Studies Depression Scale (CES-D), which asks: "During the past week, did you feel you could not get going?" Fatigue was coded as present if participants reported experiencing this feeling occasionally (3–4 days per week) or most of the time (5–7 days per week). Resistance was defined by self-reported difficulty climbing 10 stairs without resting. Ambulation was determined by whether participants reported difficulty walking a quarter of a mile. Illness burden was assessed based on self-reported physician diagnoses of 11 conditions: hypertension, diabetes, cancer, chronic lung disease, myocardial infarction, congestive heart failure, angina, asthma, arthritis, stroke, and kidney disease. The presence of five or more conditions was coded as meeting the illness criterion. Weight loss was assessed via item 2 of the CES-D: "During the past week, did you not feel like eating or have a poor appetite?" This domain was considered present if participants reported the symptom occasionally (3–4 days) or most of the time (5–7 days). Each present domain contributed one point to the total FRAIL score, resulting in a range from 0 (no components present) to 5 (all components present). Scores were categorized as follows: frail (3–5), pre-frail (1–2), and not frail (0). For binary classification, individuals with scores of 3 or higher were considered frail, while those with scores of 0–2 were considered not frail. Inclusion in the analytic sample required complete data on at least three of the five FRAIL components, consistent with criteria used in the frailty phenotype framework.

## APPENDIX III. EPIGENEITC AGE ACCELERATION

### Health and Retirement Study (HRS)

Epigenetic data were obtained from a subsample of 4,018 HRS participants, with high-quality DNA methylation (DNAm) profiles successfully generated for over 97% of samples. DNAm was assessed using the Illumina Infinium MethylationEPIC BeadChip array. To align with NHANES and other cohort studies, five widely used epigenetic clocks were selected: Horvath, Hannum, Levine PhenoAge, GrimAge, and Dunedin Pace of Aging (DunedinPoAM). For the first four clocks, epigenetic age acceleration (EAA) was calculated by regressing DNAm age on chronological age using a linear model, with the residuals representing the component of biological age not explained by chronological age. These residuals capture accelerated or decelerated aging and may range from fractions of a year to several years. As DunedinPoAm directly quantifies the pace of aging, no transformation was applied. All five clock-derived measures were standardized (z-scored), and extreme outliers were excluded prior to analysis.

*Sources*: https://hrsdata.isr.umich.edu/data-products/epigenetic-clocks and (2)

### National Health and Nutrition Examination Survey (NHANES)

Detailed documentation for DNAm and epigenetic clock data in NHANES is available at: https://wwwn.cdc.gov/nchs/nhanes/dnam/. DNAm was measured using the Illumina MethylationEPIC BeadChip array among adults aged 50 years and older from diverse demographic backgrounds, with data available for the 1999–2000 and 2001–2002 waves. The DNAm data underwent extensive preprocessing and normalization, including quality control procedures to remove sample outliers and mismatches. Available datasets include normalized methylation matrices, biomarker data, and estimated blood cell proportions. As in HRS, we selected five clocks—Horvath, Hannum, PhenoAge, GrimAge, and DunedinPoAm—and applied the same residual-based method to calculate EAA for the first four clocks, while using DunedinPoAm as-is to capture aging rate.

*Source*: https://wwwn.cdc.gov/nchs/nhanes/dnam/

### Healthy Aging in Neighborhoods of Diversity across the Life Span (HANDLS)

In the HANDLS study, DNAm was measured from peripheral blood samples using the Illumina Infinium MethylationEPIC BeadChip, consistent with the platforms used in HRS and NHANES. Epigenetic age was estimated using the Horvath and Hannum clocks, and EAA was derived by comparing the predicted DNAm age to chronological age using the residual approach. These residuals served as indicators of accelerated or decelerated biological aging. Additionally, HANDLS implemented the DunedinPACE clock to directly assess the pace of aging, in place of DunedinPoAm. This harmonized approach facilitated cross-cohort comparisons of biological aging trajectories.

*Sources:* (3, 4, 5, 6)

## APPENDIX IV. DISCRETE TIME HAZARD MODEL:

Discrete-time hazard models are statistical methods used to analyze time-to-event data when time is measured in distinct intervals (e.g., years, months, or days). These models are particularly valuable in fields like public health, social sciences, and education, where time is often recorded in discrete units. A key feature of discrete-time hazard models is their reliance on a binary logistic regression framework, allowing for flexible incorporation of time-varying covariates and straightforward estimation using standard regression software. These models offer several advantages, including effective handling of tied

event times, adaptability to varying covariate values across time, and compatibility with widely used software platforms such as R, Stata, and SAS.

In implementation, the data must be structured in a person-period format, where each individual contributes multiple records—one for each time interval at risk. The model involves defining discrete time periods, specifying a baseline hazard function (often using time dummies), and incorporating relevant covariates. Importantly, discrete-time hazard models align well with additive Bayesian network (ABN) frameworks, which support Gaussian, binomial, and Poisson distributions (see Appendix V). However, limitations include the need to discretize continuous time, potential loss of information with wide intervals, and the assumption of constant risk within each interval.

$$\log\left(\frac{h_t}{1-h_t}\right) = \beta_0 + \sum_{i=1}^{p} \beta_i X_i$$

*Sources*: (7)

## APPENDIX V. ADDITIVE BAYESIAN NETWORKS:

### A) Theoretical framework

Additive Bayesian Networks (ABNs) are a class of probabilistic graphical models that represent conditional dependencies among variables using a directed acyclic graph (DAG). They offer key benefits such as multivariate analysis, potential for causal inference, and adaptability to various data types and distributions. In ABNs, each node's distribution is modeled locally—typically using linear regression for continuous variables and logistic regression for binary outcomes. Central to ABNs is Bayes' Theorem, which guides the estimation of posterior distributions by combining the likelihood from observed data with prior distributions. The model selection process involves a scoring function, often incorporating the Bayesian Information Criterion (BIC), to balance fit and complexity. ABNs are widely applied in disciplines like epidemiology, genetics, and the social sciences to examine complex inter-variable relationships and uncover potential causal pathways.

*Sources*: (8, 9)

The following set of equations are used in this method:

**(Eq. 1.1)** *Linear regression*: $Y = \beta_0 + \sum_{i=1}^{k} \beta_i X_i + \varepsilon$

**(Eq. 1.2)** *Logistic regression*: $logit\big(P(Y=1|X_1, \ldots, X_k)\big) = \beta_0 + \sum_{i=1}^{k} \beta_i X_i$

**(Eq. 1.3)** *Likelihood Function*: $\boldsymbol{L(\theta|D) = \prod_{i=1}^{n} P(X_i \mid Parents(X_i), \theta_i)}$

**(Eq. 1.4)** *Bayesian Posterior*: $\boldsymbol{P(\theta|D) = \frac{P(D|\theta)P(\theta)}{P(D)}}$

**(Eq. 3.5)** *BIC for Model Selection*: $\boldsymbol{BIC = -2log\big(L(\theta|D)\big) + p \times log(n)}$

**B) Additive Bayesian Network workflow**

The R code, available on GitHub and applied to both NHANES and HRS datasets, provides a comprehensive framework for conducting ABN analysis. This includes steps for software installation, data preprocessing, specification of structural constraints, model fitting, and iterative model refinement. To determine the optimal number of parent nodes for each child node, the study assessed the stabilization of the log marginal likelihood in relation to model complexity across key variables. Given the high computational demands of ABN modeling and the large analytic sample (e.g., over 18,000 person-period observations in NHANES 1999–2019), the maximum number of parents per child node was limited to three. A two-parent configuration was only retained when the log marginal likelihood showed clear stabilization compared to the three-parent model.

*Source*: https://r-bayesian-networks.org/

## APPENDIX VI. GENERALIZED STRUCTURAL EQUATIONS MODELS

Stata's Generalized Structural Equation Modeling (gsem) framework provides a versatile approach for modeling both linear and nonlinear relationships among variables. It supports a wide range of outcome types and allows for the inclusion of random effects. Estimation is conducted using Maximum Likelihood or Quasi-Maximum Likelihood methods, and model fit is assessed through various goodness-of-fit statistics and tests. The framework also enables users to generate linear predictions, compute residuals, and evaluate both linear and nonlinear combinations of model parameters.

**Probability density function for Weibull distribution**

$$f(t;\delta,k) = \frac{k}{\delta}(\frac{t}{\delta})^{k-1}e^{-(\frac{t}{\delta})^k}, t \geq 0$$

*Where:*

- *t is the time or random variable of interst.*
- *δ>0 is the scale parameter*
- *k>0 is the shape parameter*

**Cumulative distribution function (CDF) and survival function (complement of CDF) for Weibull distribution**

$$F(t;\delta,k) = 1 - e^{-(\frac{t}{\delta})^k}, t \geq 0$$

$$S(t;\delta,k) = e^{-(\frac{t}{\delta})^k}, t \geq 0$$

**Hazard function for Weibull distribution**

$$h(t;\delta,k) = \frac{f(t;\delta,k)}{S(t;\delta,k)} = \frac{k}{\delta}\left(\frac{t}{\delta}\right)^{k-1}, t \geq 0$$

*Source*: https://www.stata.com/manuals/semgsem.pdf

**APPENDIX VII. Four-way decomposition models**

Causal mediation analysis seeks to quantify the pathways through which an exposure affects an outcome, distinguishing between direct and indirect effects. Traditional approaches often assume no interaction between the exposure and the mediator. To overcome these limitations, four-way decomposition extends mediation analysis by accounting for both mediation and exposure–mediator interaction, partitioning the total effect into four components:

1. Controlled Direct Effect (CDE): The portion of the effect of the exposure on the outcome that is not mediated and not due to interaction.
2. Pure Indirect Effect (PIE): The effect that is solely mediated by the mediator, assuming no interaction.
3. Mediated Interaction (INTmed): The component attributable to both mediation and interaction—i.e., the mediator influences the outcome, and its effect depends on exposure.
4. Reference Interaction (INTref): The component that arises solely due to interaction between the exposure and mediator, assuming the mediator is fixed at a reference level.

To estimate these components, we used the med4way command in Stata with the *fulloutput* option. This provided the following parameters:

- tereri: *Total Excess Relative Risk* — the excess relative risk (RR - 1) comparing exposed vs. unexposed.
- terira: *Total Effect Risk Ratio* — the RR comparing the exposed group to the unexposed.
- ereri_cde: *Excess RR due to Controlled Direct Effect* — portion of the effect not explained by mediation or interaction.
- ereri_pie: *Excess RR due to Pure Indirect Effect* — mediated effect assuming no interaction.
- ereri_intmed: *Excess RR due to Mediated Interaction* — effect due to joint mediation and interaction.
- ereri_intref: *Excess RR due to Reference Interaction* — effect due solely to interaction with the mediator fixed at the reference level.

We also report proportions:

- p_cde: Proportion of the total effect attributable to the controlled direct effect (ereri_cde / tereri).
- p_pie: Proportion due to the pure indirect effect.
- p_intmed: Proportion due to mediated interaction.
- p_intref: Proportion due to reference interaction.

Global effect summaries include:

- op_m: *Overall Proportion Mediated* — the combined proportion mediated through PIE and INTmed.
- op_ati: *Overall Proportion Attributable to Interaction* — combined contribution of INTmed and INTref.
- op_e: *Overall Proportion Eliminated* — the proportion of the total effect that would be removed if the mediator were fixed at the reference level.

**Assumptions**

This decomposition relies on several key assumptions:

- No unmeasured confounding between exposure–outcome, exposure–mediator, and mediator–outcome paths.
- Correct model specification for both outcome and mediator.
- Positivity (non-zero probability of all covariate combinations).

- Consistency (observed data reflect counterfactual outcomes under the same conditions).

1. Counterfactual notation
Let:
- $A \in \{0,1\}$= exposure
- $M \in \{0,1\}$= mediator
- $Y$= outcome
- $Y_{a,m}$= potential outcome if $A = a, M = m$
- $M_a$= mediator value if $A = a$

Total effect (risk ratio scale):

$$RR_{TE} = \frac{E[Y_{1,M_1}]}{E[Y_{0,M_0}]}$$

Excess relative risk:

$$TERERI = RR_{TE} - 1$$

2. Four-way decomposition (Excess RR scale)
The total excess relative risk decomposes as:

$$TERERI = ERERI_{CDE} + ERERI_{PIE} + ERERI_{INTmed} + ERERI_{INTref}$$

(1) Controlled Direct Effect (CDE)
Mediator fixed at reference level $m = 0$:

$$ERERI_{CDE} = \frac{E[Y_{1,0}]}{E[Y_{0,0}]} - 1$$

(2) Pure Indirect Effect (PIE)
Effect of mediator under no interaction:

$$ERERI_{PIE} = \frac{E[Y_{0,M_1}]}{E[Y_{0,M_0}]} - 1$$

(3) Mediated Interaction (INTmed)
Joint mediation and interaction component:

$$ERERI_{INTmed} = \frac{E[Y_{1,M_1}] - E[Y_{1,M_0}] - E[Y_{0,M_1}] + E[Y_{0,M_0}]}{E[Y_{0,M_0}]}$$

(4) Reference Interaction (INTref)
Interaction when mediator fixed at reference:

$$ERERI_{INTref} = \frac{E[Y_{1,M_0}] - E[Y_{0,M_0}] - \left(E[Y_{1,M_1}] - E[Y_{0,M_1}]\right)}{E[Y_{0,M_0}]}$$

3. Total Effect Risk Ratio

$$TERIRA = RR_{TE} = \frac{E[Y_{1,M_1}]}{E[Y_{0,M_0}]}$$
$$TERERI = TERIRA - 1$$

4. Proportions

$$p_{CDE} = \frac{ERERI_{CDE}}{TERERI}$$
$$p_{PIE} = \frac{ERERI_{PIE}}{TERERI}$$
$$p_{INTmed} = \frac{ERERI_{INTmed}}{TERERI}$$
$$p_{INTref} = \frac{ERERI_{INTref}}{TERERI}$$

5. Global summaries

Overall Proportion Mediated

$$op_m = \frac{ERERI_{PIE} + ERERI_{INTmed}}{TERERI}$$

Overall Proportion Attributable to Interaction

$$op_{ati} = \frac{ERERI_{INTmed} + ERERI_{INTref}}{TERERI}$$

Overall Proportion Eliminated (if mediator fixed at reference)

$$op_e = \frac{ERERI_{PIE} + ERERI_{INTmed} + ERERI_{INTref}}{TERERI}$$

Assumptions (formalized)

1. No unmeasured confounding

$$Y_{a,m} \perp A \mid C$$
$$M_a \perp A \mid C$$
$$Y_{a,m} \perp M \mid A, C$$

2. Positivity

$$0 < P(A = a \mid C) < 1$$

3. Consistency

$$Y = Y_{a,m} \text{ if } A = a, M = m$$

4. Correct model specification

Outcome and mediator models correctly specified.

**Implementation**

Models were specified using Cox regression for time-to-event outcomes and linear regression for continuous mediators. Confidence intervals were computed using the delta method. The results provide nuanced causal insights, distinguishing how much of the effect is mediated biologically (e.g., through epigenetic age acceleration) and how much is due to direct or interactive pathways.

*Sources*: (10, 11)

**Sensitivity analyses controlling for WBC composition:**

For harmonized analyses of leukocyte composition, comparable complete blood count (CBC) variables are available from NHANES 1999–2002 and the Health and Retirement Study (HRS) 2016 Venous Blood Study (VBS). NHANES CBC documentation is available at:
1999–2000: https://wwwn.cdc.gov/Nchs/Data/Nhanes/Public/1999/DataFiles/LAB25.htm
2001–2002: https://wwwn.cdc.gov/Nchs/Data/Nhanes/Public/2001/DataFiles/L25_B.htm

In NHANES 1999–2002, venous whole blood was collected in Mobile Examination Centers and analyzed using standardized automated hematology analyzers under strict quality-control protocols. The CBC panel includes total white blood cell (WBC) count and a five-part differential reported as both percentages and absolute counts. The percentage variables include neutrophils, lymphocytes, monocytes, eosinophils, and basophils (each expressed as percent of total WBC). Absolute counts for each subtype (cells $\times 10^3/\mu L$) are also provided. For harmonization focused on leukocyte composition adjustment, the key variables are the five differential percentages and total WBC count, which together capture circulating immune cell distribution independent of red cell or platelet parameters. These measures are widely used to control for immune heterogeneity in analyses of inflammation, epigenetic aging, gene expression, and other molecular phenotypes.

The HRS 2016 Venous Blood Study (VBS) provides directly comparable venous-based CBC measures collected by trained phlebotomists during in-home visits and analyzed in certified laboratories using automated hematology platforms. Documentation is available through the HRS biomarker and VBS data description pages: https://hrsdata.isr.umich.edu/documentation (see 2016 Venous Blood Study files). The HRS VBS CBC includes total WBC count and a five-part differential, reported as both percentages and absolute counts for neutrophils, lymphocytes, monocytes, eosinophils, and basophils. Because both NHANES and HRS VBS use venous blood and automated differential counting, the percentage distributions of leukocyte subtypes are conceptually and methodologically comparable across studies. For harmonized modeling, the primary shared variables are: total WBC count and percent neutrophils, percent lymphocytes, percent monocytes, percent eosinophils, and percent basophils. These percentage measures provide a standardized representation of leukocyte composition suitable for covariate adjustment to reduce confounding due to variation in circulating immune cell mixtures. Using percentages rather than absolute counts further enhances cross-cohort comparability when laboratory platforms or calibration standards differ slightly between surveys.

Thus, as sensitivity analysis and in both NHANES 1999-2002 and HRS 2016, WBC composition was entered into the four-way composition models by including several common WBC percentage by sub-type into the model as exogenous variables in addition to the covariates that were originally entered into

the model. The exact list of covariates that is finally included in the sensitivity analyses is provided in the main methods section.

**Sensitivity analyses for bi-directional relationships: Epigenetic aging → Frailty → mortality**

To evaluate potential reverse or bi-directional pathways, we conducted sensitivity analyses specifying epigenetic aging as the exposure, frailty as the mediator, and mortality as the outcome. This framework tests whether accelerated biological aging increases mortality risk indirectly through increased frailty burden, while also allowing for exposure–mediator interaction. Using the same four-way decomposition approach implemented via the *med4way* command in Stata, we partitioned the total effect of epigenetic age acceleration on mortality into the Controlled Direct Effect (CDE), Pure Indirect Effect (PIE), Mediated Interaction (INTmed), and Reference Interaction (INTref). This allowed us to distinguish whether frailty functions primarily as a downstream pathway linking epigenetic aging to mortality, or whether the mortality risk associated with epigenetic aging operates largely independently of frailty status.

Models were specified consistently with the primary analysis, using Cox regression for mortality and linear regression for frailty indices, adjusting for demographic, socioeconomic, and health-related covariates. We compared the overall proportion mediated (op_m) and proportion attributable to interaction (op_ati) across forward and reverse models to assess directional robustness. If the mediated proportion remained substantial under this reversed specification, it would support a bidirectional feedback mechanism between biological aging and clinical vulnerability. Conversely, attenuation of the mediated component would suggest that epigenetic aging more plausibly operates downstream of frailty rather than as its upstream determinant.

Below are the **equations rewritten explicitly for the reverse-causation specification**:

- **Exposure:** epigenetic aging metric $A$(continuous; for interpretation you can set $A = a_1$vs $A = a_0$, e.g., +1 SD vs 0)
- **Mediator:** frailty index $M$(continuous)
- **Outcome:** mortality time-to-event $T$(Cox model), with effect summarized on an **RR/HR scale** (as in med4way output)

**1) Counterfactual definitions (reverse model)**

Let:

- $M_a$= frailty burden that would be observed if epigenetic aging were set to $A = a$
- $Y_{a,m}$= potential mortality outcome if $A = a$ and frailty were set to $M = m$
  (with $E[Y.]$interpreted as a **risk** or **hazard-based risk ratio** target used by med4way)

Total effect (epigenetic aging → mortality, through all pathways including frailty):

$$RR_{TE} \equiv TERIRA = \frac{E\left[Y_{a_1,M_{a_1}}\right]}{E\left[Y_{a_0,M_{a_0}}\right]}$$

Total excess relative risk:

$$TERERI = TERIRA - 1$$

**2) Four-way decomposition (reverse model; Excess RR scale)**

$$TERERI = ERERI_{CDE} + ERERI_{PIE} + ERERI_{INTmed} + ERERI_{INTref}$$

Choose a **reference frailty level** m(often $m = 0$ if $M$is centered/standardized; otherwise a meaningful baseline).

**(1) Controlled Direct Effect (CDE)**

Effect of epigenetic aging on mortality **holding frailty fixed** at $m$ :

$$ERERI_{CDE} = \frac{E\left[Y_{a_1, m}\right]}{E\left[Y_{a_0, m}\right]} - 1$$

**(2) Pure Indirect Effect (PIE)**

Effect of epigenetic aging on mortality **only through changing frailty**, evaluated at baseline exposure $a_0$:

$$ERERI_{PIE} = \frac{E\left[Y_{a_0, M_{a_1}}\right]}{E\left[Y_{a_0, M_{a_0}}\right]} - 1$$

**(3) Mediated Interaction (INTmed)**

Portion due to **both** mediation and exposure–mediator interaction:

$$ERERI_{INTmed} = \frac{E\left[Y_{a_1, M_{a_1}}\right] - E\left[Y_{a_1, M_{a_0}}\right] - E\left[Y_{a_0, M_{a_1}}\right] + E\left[Y_{a_0, M_{a_0}}\right]}{E\left[Y_{a_0, M_{a_0}}\right]}$$

**(4) Reference Interaction (INTref)**

Portion due to **interaction only**, when frailty is set to what it would be under $a_0$:

$$ERERI_{INTref} = \left( \frac{E\left[Y_{a_1, M_{a_0}}\right]}{E\left[Y_{a_0, M_{a_0}}\right]} - \frac{E\left[Y_{a_1, m}\right]}{E\left[Y_{a_0, m}\right]} \right)$$

(Equivalent to "interaction contribution beyond the CDE" on the excess-RR scale.)

**3) Proportions (reverse model)**

$$p_{CDE} = \frac{ERERI_{CDE}}{TERERI}, p_{PIE} = \frac{ERERI_{PIE}}{TERERI}, p_{INTmed} = \frac{ERERI_{INTmed}}{TERERI}, p_{INTref} = \frac{ERERI_{INTref}}{TERERI}$$

**4) Global summaries used for directionality checks**

Overall proportion mediated (frailty-mediated share):

$$op_m = \frac{ERERI_{PIE} + ERERI_{INTmed}}{TERERI}$$

Overall proportion attributable to interaction (synergism share):

$$op_{ati} = \frac{ERERI_{INTmed} + ERERI_{INTref}}{TERERI}$$

Overall proportion eliminated if frailty were fixed at m:

$$op_e = \frac{ERERI_{PIE} + ERERI_{INTmed} + ERERI_{INTref}}{TERERI}$$

Interpretation in **reverse causation** language:

- **Large** $op_m \Rightarrow$ epigenetic aging affects mortality partly through increasing frailty burden (supporting an upstream role of biological aging).

- Large $op_{ati}$⇒ synergism: the mortality effect of frailty differs by epigenetic aging level (or vice versa).
- Attenuated $op_m$relative to the forward model ⇒ frailty is less plausible as a downstream mediator of epigenetic aging (more consistent with epigenetic aging operating downstream of frailty or reflecting shared causes).

**5) Model specification**
Mediator model (logistic regression):

Let $A$be the epigenetic aging metric (continuous, e.g., SD units), $M \in \{0,1\}$be **frailty status**, and $C$covariates.

$$\text{logit}\{P(M = 1 \mid A, C)\} = \alpha_0 + \alpha_A A + \alpha_C^\top C$$

Equivalently,

$$P(M = 1 \mid A, C) = \frac{\exp(\alpha_0 + \alpha_A A + \alpha_C^\top C)}{1 + \exp(\alpha_0 + \alpha_A A + \alpha_C^\top C)}$$

Outcome model (Cox with interaction):

Mortality hazard given $A$, $M$, and $C$:

$$h(t \mid A, M, C) = h_0(t)\exp(\beta_A A + \beta_M M + \beta_{AM}(A \cdot M) + \beta_C^\top C)$$

This implies the **hazard ratio for a one-unit increase in** $A$depends on frailty status:

- Among non-frail ($M = 0$):

$$HR_{A|M=0} = \exp(\beta_A)$$

- Among frail ($M = 1$):

$$HR_{A|M=1} = \exp(\beta_A + \beta_{AM})$$

And the **effect of frailty on mortality** depends on epigenetic aging:

$$HR_{M|A} = \exp(\beta_M + \beta_{AM} A)$$

**6) Assumptions (reverse model; stated for this direction)**
No unmeasured confounding (conditional on covariates $C$):

$$Y_{a,m} \perp A \mid C, M_a \perp A \mid C, Y_{a,m} \perp M \mid A, C$$

Positivity:

$$0 < f(A \mid C) \text{ on the support used (e.g., } a_0, a_1)$$

Consistency:

$$M = M_a \text{ if } A = a, Y = Y_{a,m} \text{ if } A = a, M = m$$

Correct specification of the mediator and Cox models.

## APPENDIX VIII. Sensitivity analysis findings

### Sensitivity analysis 1: adjustment for WBC composition

#### NHANES

In NHANES, the primary analysis (frailty → mortality, EAA as mediator) shows that the total effect (TE) of frailty on mortality remains statistically significant across clocks and is only modestly attenuated after WBC adjustment. Mediation is clock-specific: Horvath and Hannum show little evidence of a pure indirect effect (PIE), whereas DunedinPoAm, PhenoAge, and especially GrimAge demonstrate significant mediation that persists after WBC adjustment, although somewhat attenuated. GrimAge consistently exhibits the largest mediated component. The controlled direct effect (CDE) remains the dominant contributor to the TE across models, indicating that frailty's association with mortality is largely independent of epigenetic aging.

Interaction components (INTmed and INTref) are generally small. INTmed is modestly positive for DunedinPoAm, PhenoAge, and GrimAge, suggesting limited amplification when frailty and accelerated aging co-occur, while INTref is negligible. Importantly, these interaction patterns are not materially altered by WBC adjustment. However, caution is warranted in interpreting differences between WBC-adjusted and unadjusted models because WBC-adjusted analyses rely on smaller analytic samples. Some attenuation of mediation or interaction components may reflect reduced statistical power rather than true biological change.

#### HRS

In HRS, patterns are broadly similar but more sensitive to WBC adjustment. In primary models (frailty → mortality, EAA mediates), TE remains significant across clocks but attenuates more noticeably after WBC adjustment. GrimAge shows the most consistent mediation signal, remaining significant after adjustment. DunedinPoAm mediation weakens substantially with WBC adjustment, and PhenoAge, Hannum, and Horvath show minimal mediation. The CDE accounts for most of the TE in all models. Interaction components are generally small and unstable. INTmed is occasionally positive for GrimAge and DunedinPoAm in unadjusted models but attenuates after WBC adjustment. INTref remains minimal. However, these differences must be interpreted cautiously because WBC-adjusted models are based on smaller subsamples, reducing precision and potentially obscuring modest mediated or interaction effects.

### Sensitivity analysis 2A: reverse causation

In NHANES reverse-causation models (epigenetic aging → frailty → mortality), second- and third-generation clocks showed the most consistent evidence of mediation. Horvath EAA demonstrated minimal total effect and no meaningful mediation. Hannum and PhenoAge showed significant pure indirect effects, though the overall proportion mediated was modest and borderline for PhenoAge. DunedinPoAm showed a stronger total association (RR≈1.39), with approximately 10% mediated through frailty and evidence of positive mediated interaction. GrimAge exhibited the largest total effect (RR≈1.66), with roughly 11% mediated and some contribution from interaction components. Overall, frailty explained about 7–11% of the total association for more advanced clocks.

In HRS, findings were similar but generally stronger. Horvath again showed no meaningful total effect or mediation. In contrast, Hannum, PhenoAge, DunedinPoAm (RR≈1.51), and GrimAge demonstrated significant total effects, with approximately 8–18% of associations mediated through frailty. Interaction components contributed modestly for DunedinPoAm and GrimAge. Across cohorts, frailty accounted for a consistent but partial share of the epigenetic aging–mortality association.

**Sensitivity analysis 2B: reverse causation + WBC adjustment**

**NHANES**

In reverse-causation models (EAA → mortality, frailty mediates), TE remains significant for second-generation clocks and is only slightly attenuated after WBC adjustment. Frailty mediation persists for DunedinPoAm, PhenoAge, and GrimAge, though some weakening (e.g., Hannum) is observed. Interaction effects remain small. Overall, NHANES findings appear relatively robust to WBC adjustment, but sample-size differences between models necessitate cautious comparison, particularly for marginal mediation effects.

**HRS**

In reverse-causation models (EAA → mortality, frailty mediates), TE remains significant for Hannum, DunedinPoAm, PhenoAge, and GrimAge, though attenuated after WBC adjustment. Frailty mediation persists most consistently for GrimAge (and often PhenoAge), whereas mediation for Hannum and DunedinPoAm becomes non-significant after adjustment. Given the smaller WBC-adjusted sample, loss of statistical significance may reflect limited power rather than elimination of mediation. Overall, HRS results suggest robustness primarily for GrimAge, but comparisons between adjusted and unadjusted models should be made with caution due to sample-size differences.

## SUPPLEMENTARY REFRENCES


1. Fried LP, Tangen CM, Walston J, Newman AB, Hirsch C, Gottdiener J, et al. Frailty in older adults: evidence for a phenotype. J Gerontol A Biol Sci Med Sci. 2001;56(3):M146-56.
2. Beydoun MA, Beydoun HA, Noren Hooten N, Maldonado AI, Weiss J, Evans MK, et al. Epigenetic clocks and their association with trajectories in perceived discrimination and depressive symptoms among US middle-aged and older adults. Aging (Albany NY). 2022;14(13):5311-44.
3. Evans MK, Lepkowski JM, Powe NR, LaVeist T, Kuczmarski MF, Zonderman AB. Healthy aging in neighborhoods of diversity across the life span (HANDLS): overcoming barriers to implementing a longitudinal, epidemiologic, urban study of health, race, and socioeconomic status. Ethn Dis. 2010;20(3):267-75.
4. Beydoun MA, Hossain S, Chitrala KN, Tajuddin SM, Beydoun HA, Evans MK, et al. Association between epigenetic age acceleration and depressive symptoms in a prospective cohort study of urban-dwelling adults. J Affect Disord. 2019;257:64-73.
5. Beydoun MA, Shaked D, Tajuddin SM, Weiss J, Evans MK, Zonderman AB. Accelerated epigenetic age and cognitive decline among urban-dwelling adults. Neurology. 2020;94(6):e613-e25.
6. Belsky DW, Caspi A, Corcoran DL, Sugden K, Poulton R, Arseneault L, et al. DunedinPACE, a DNA methylation biomarker of the pace of aging. Elife. 2022;11.
7. Kvamme H, Borgan O. Continuous and discrete-time survival prediction with neural networks. Lifetime Data Anal. 2021;27(4):710-36.
8. Lewis FI, Ward MP. Improving epidemiologic data analyses through multivariate regression modelling. Emerg Themes Epidemiol. 2013;10(1):4.
9. Scutari M, Denis, J.-B.,. Bayesian Networks With Examples in R. Boca Raton, FL: CRC Press; 2022.
10. VanderWeele TJ, Shrier I. Sufficient Cause Representation of the Four-way Decomposition for Mediation and Interaction. Epidemiology. 2016;27(5):e32-3.

11. Discacciati A, Bellavia A, Lee JJ, Mazumdar M, Valeri L. Med4way: a Stata command to investigate mediating and interactive mechanisms using the four-way effect decomposition. Int J Epidemiol. 2018.
