# Supplementary material for: Epigenetic aging markers in the association between frailty and mortality among U.S. adults
Source: BMC Med. 2026 Apr 15;24:323. doi: 10.1186/s12916-026-04866-0 (PMC13192009; doi:10.1186/s12916-026-04866-0)
Supplement: Supplementary file 2 — Additional file 2: Figures S1. Fig. S1 – Participant flowcharts for NHANES (1999–2002), HRS (2016), and HANDLS (2004–2009), showing sample selection and exclusions. [file 12916_2026_4866_MOESM2_ESM.pdf]

FIGURE S1. Participant flowcharts for NHANES, HRS and HANDLS samples

(A) NHANES 1999-2002

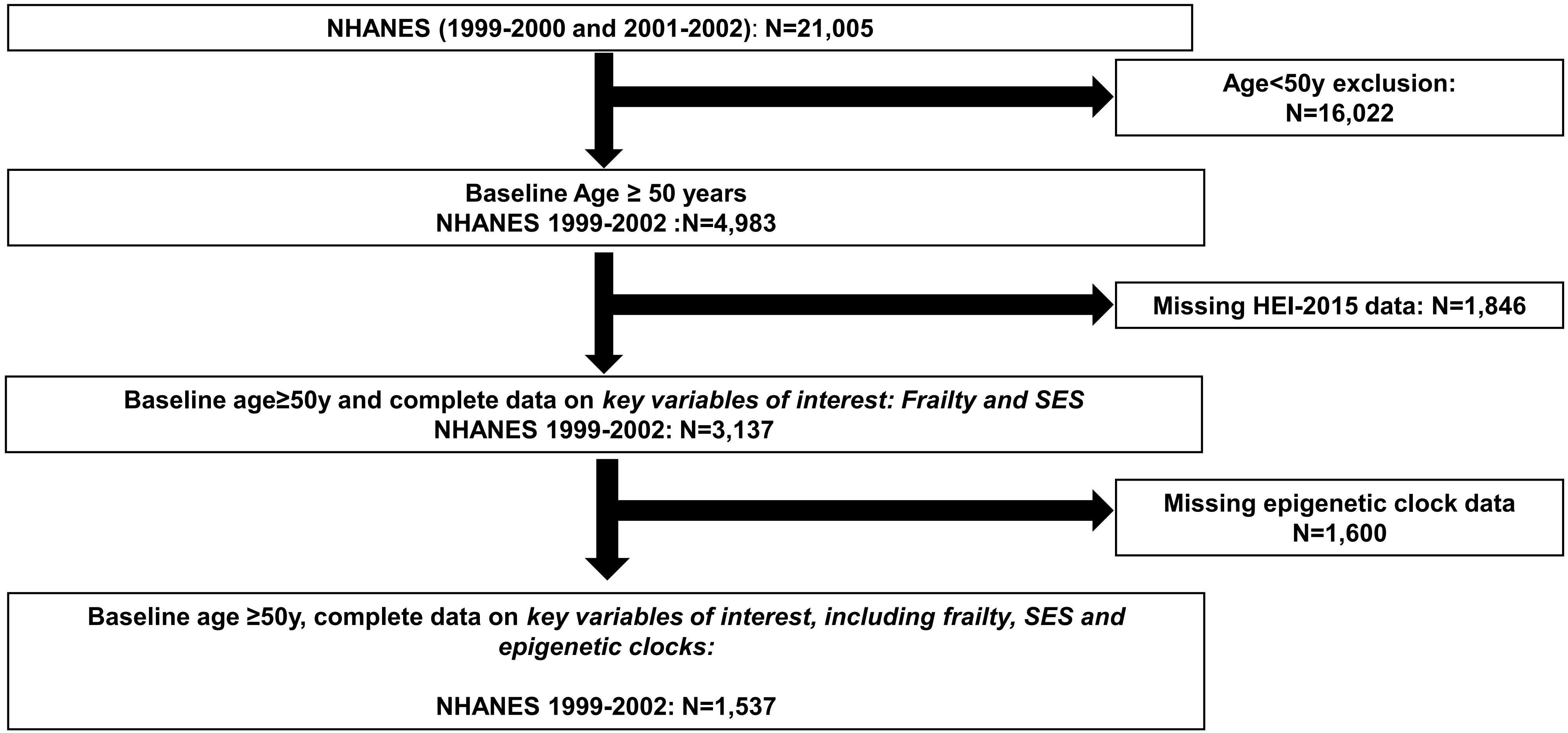

(B) HRS 2016

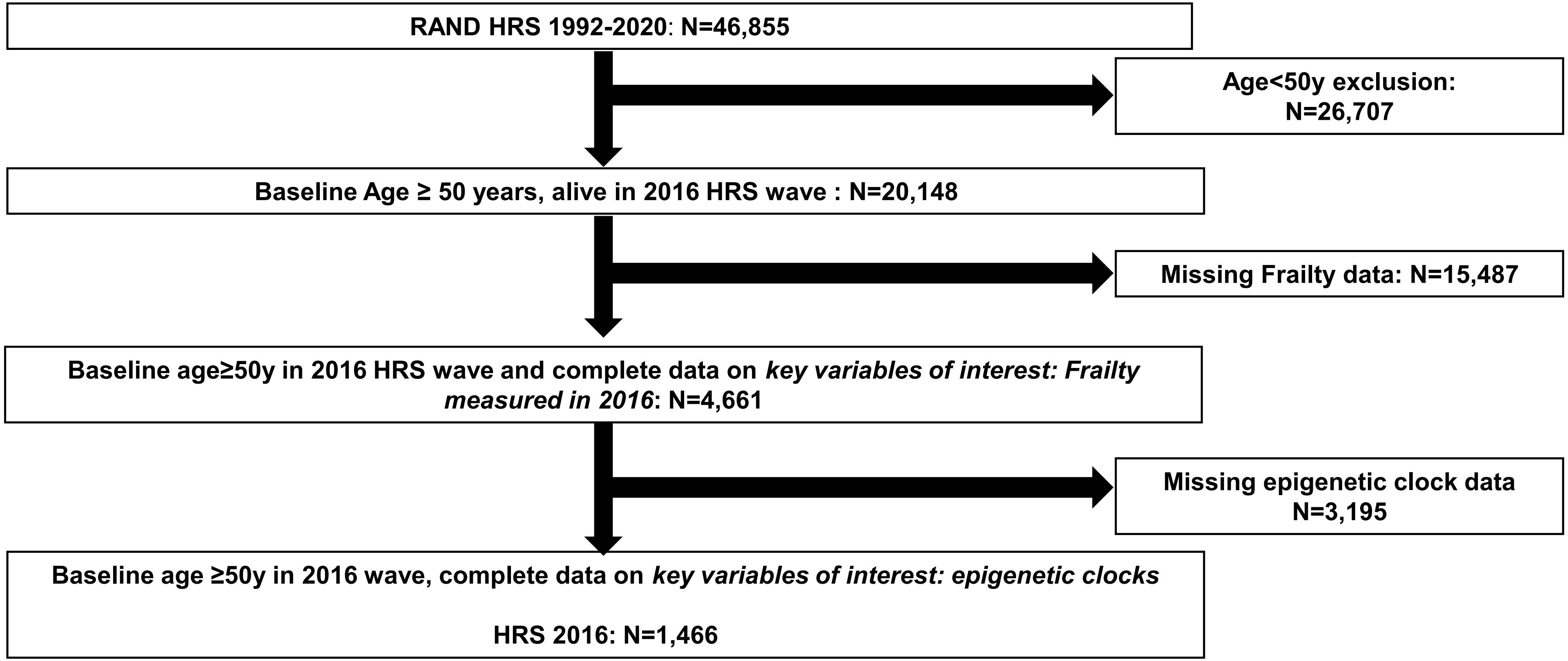
RAND HRS 1992-2020: N=46,855
Age<50y exclusion: N=26,707
Baseline Age ≥ 50 years, alive in 2016 HRS wave : N=20,148
Missing Frailty data: N=15,487
Baseline age≥50y in 2016 HRS wave and complete data on key variables of interest: Frailty measured in 2016: N=4,661
Missing epigenetic clock data N=3,195
Baseline age ≥50y in 2016 wave, complete data on key variables of interest: epigenetic clocks
HRS 2016: N=1,466

(C) HANDLS 2004-2009

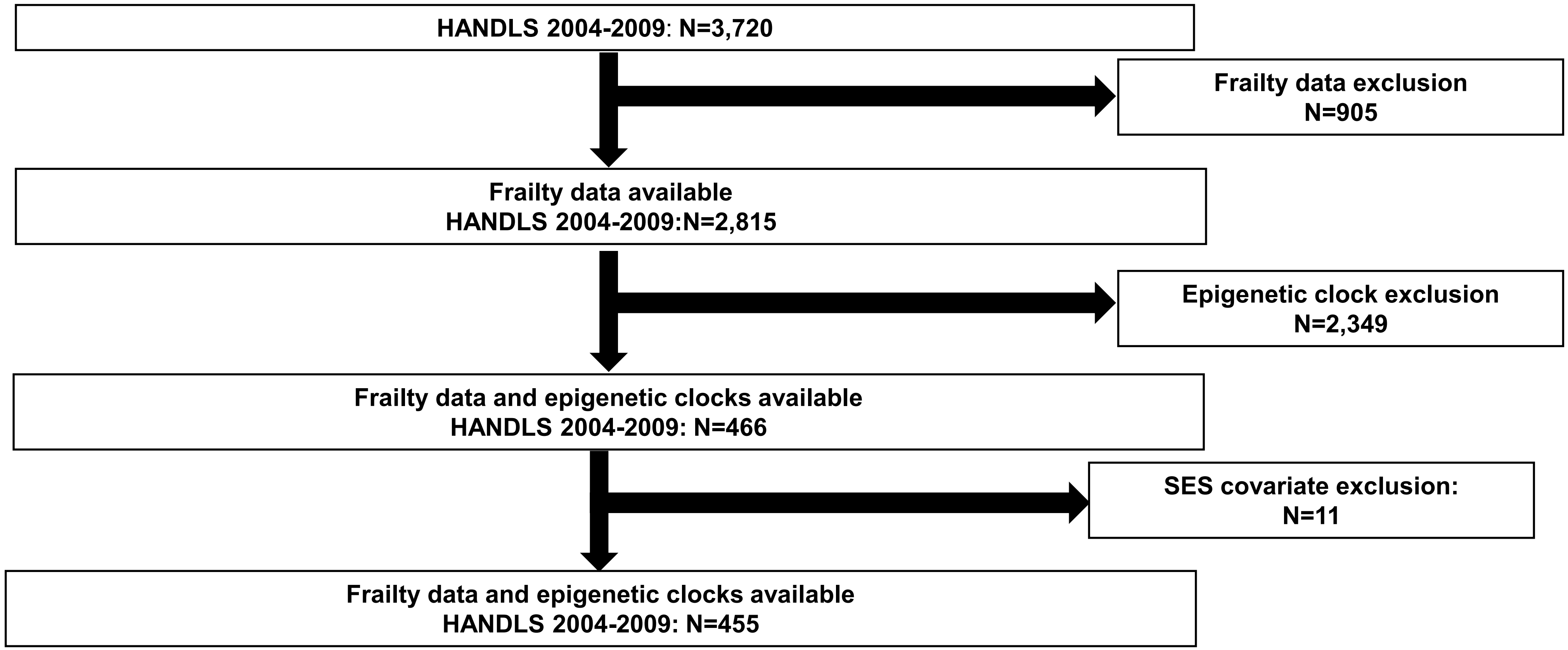

Abbreviations: HANDLS=Healthy Aging in Neighborhoods of Diversity across the Life Span; HRS=Health and Retirement Study; NHANES=National Health and Nutrition Examination Surveys.
