# Supplementary material for: Epigenetic aging markers in the association between frailty and mortality among U.S. adults
Source: BMC Med. 2026 Apr 15;24:323. doi: 10.1186/s12916-026-04866-0 (PMC13192009; doi:10.1186/s12916-026-04866-0)
Supplement: Supplementary file 3 — Additional file 3: Figure S2. Fig. S2 – Pearson correlation matrices and kernel density plots of frailty scores across NHANES, HRS, and HANDLS, illustrating relationships among SES, frailty, and epigenetic aging measures. [file 12916_2026_4866_MOESM3_ESM.pdf]

FIGURE S2. Pearson's correlation matrix between SES, frailty, and epigenetic clock metrics and kernel smoothed distribution of the frailty score: NHANES 1999–2002, HRS 2016 and HANDLS 2004-2009

(A) NHANES 1999-2002

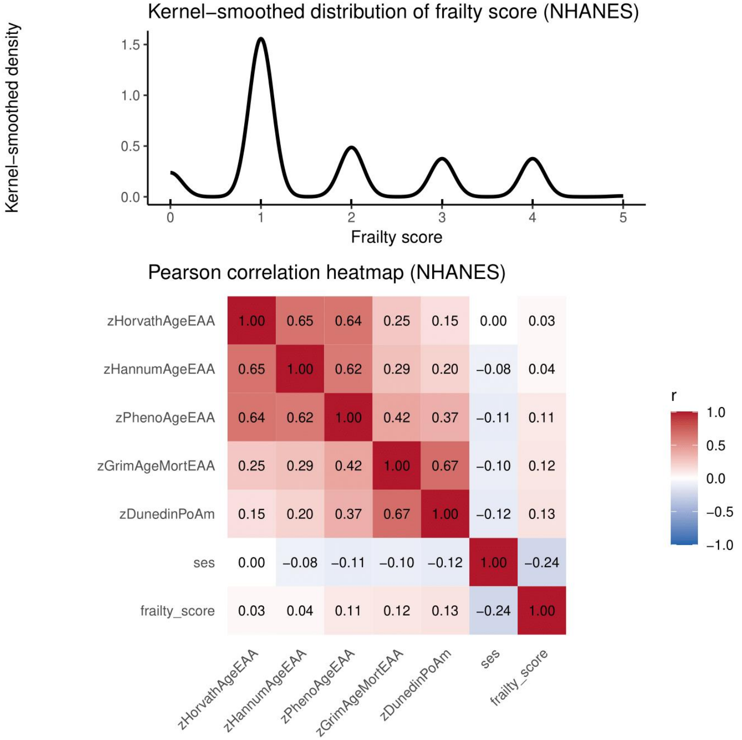

(B) HRS 2016

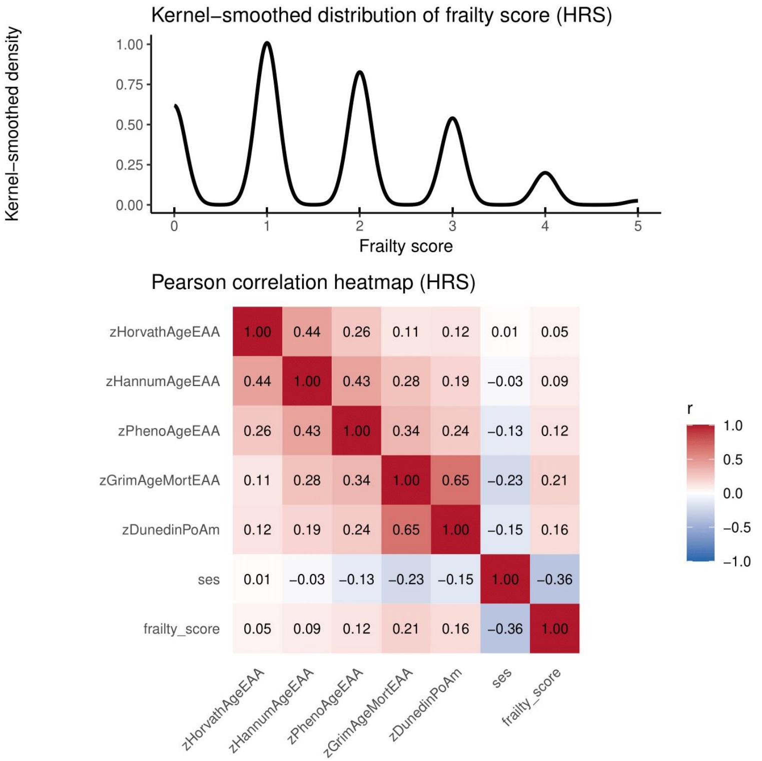

(C) HANDLS 2004-2009

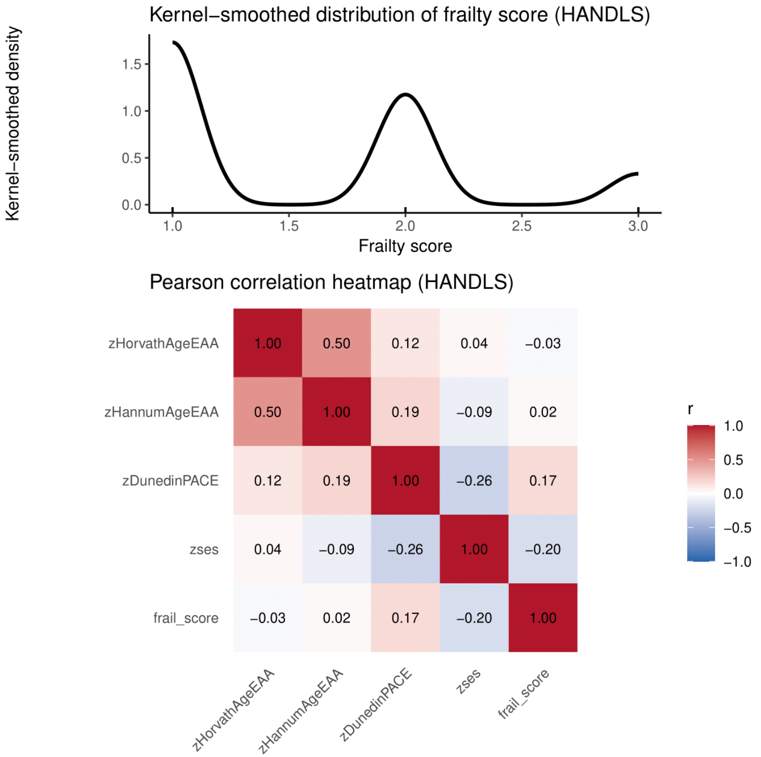

Abbreviations:
DunedinPoAm = Dunedin Pace of Aging DNA methylation clock; GrimAgeEAA = Grim DNA methylation Epigenetic Age Acceleration; HANDLS=Healthy Aging in Neighborhoods of Diversity Across the Life Span; HannumAgeEAA = Hannum DNA methylation Age, Epigenetic Age Acceleration; HorvathAgeEAA = Horvath DNA methylation Age, Epigenetic Age Acceleration; HRS = Health and Retirement Study; NHANES = National Health and Nutrition Examination Surveys; PhenoAgeEAA = Pheno DNA methylation Age Epigenetic Age Acceleration; SES = Socio-economic Status; z = standardized z-score.
Notes: Sampling weights were not accounted for in this analysis. Unweighted sample sizes were n=1,537 for NHANES, n=1,413 for HRS and n=455 for HANDLS.
