# Supplementary material for: Epigenetic aging markers in the association between frailty and mortality among U.S. adults
Source: BMC Med. 2026 Apr 15;24:323. doi: 10.1186/s12916-026-04866-0 (PMC13192009; doi:10.1186/s12916-026-04866-0)
Supplement: Supplementary file 6 — Additional file 6: Figure S3. Fig. S3 – Additive Bayesian network (ABN) models for NHANES and HRS under varying parent limits (1–3 parents per node), including final network structures and model fit comparisons across specifications. [file 12916_2026_4866_MOESM6_ESM.pdf]

FIGURE S3. ABN findings using discrete time hazards models, for 1 and 2 parents/child limits

(A) NHANES 1999-2002, follow-up till 2019

1 parent/child

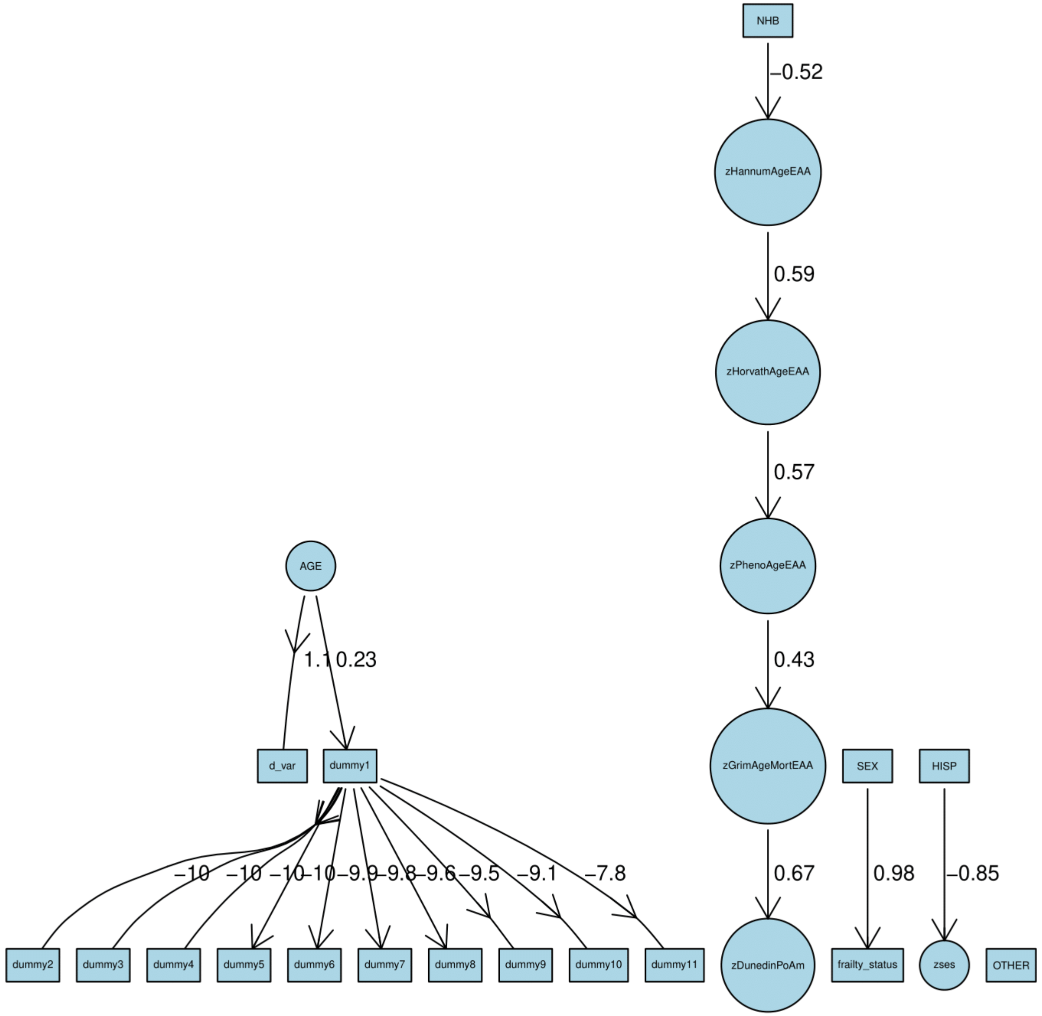

2 parents/child

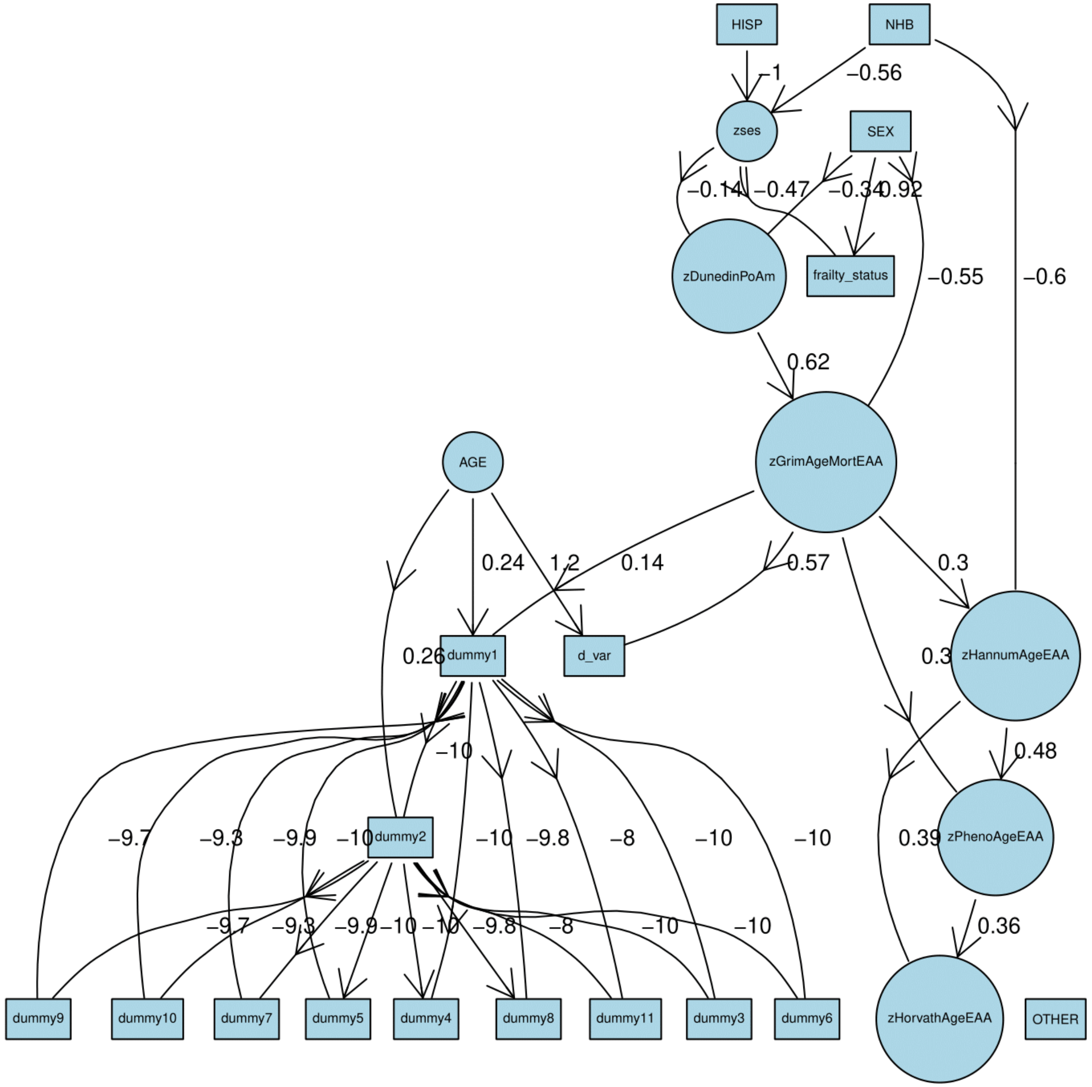

NHANES 1999-2019: Final solution

3 parents/child solution

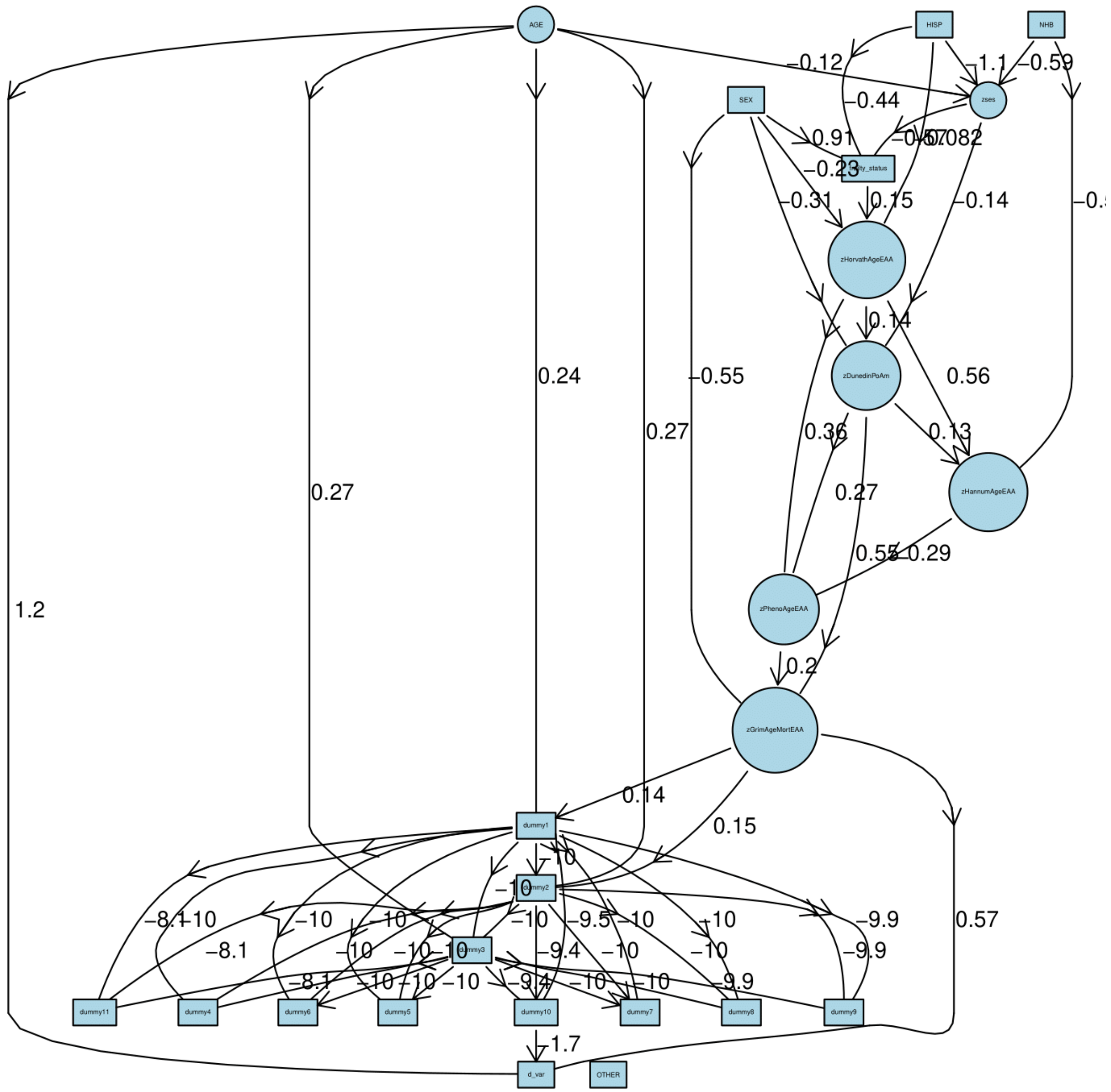

Model fit for 1-3 parents/child

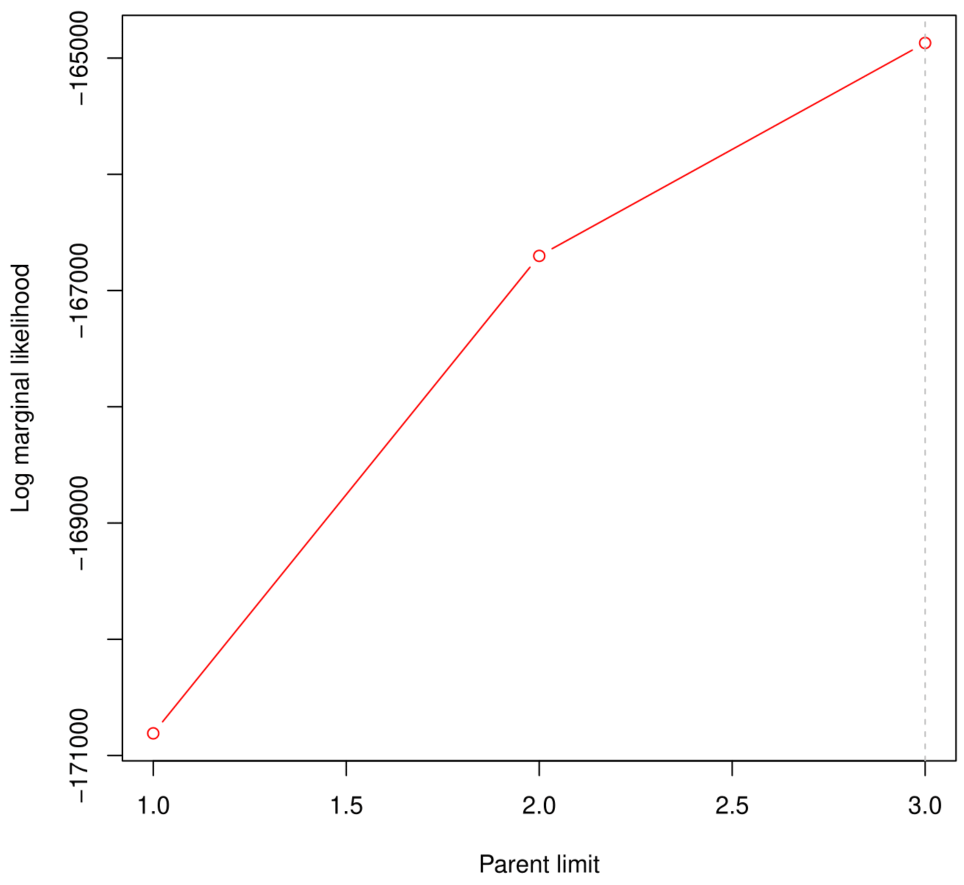

(B) HRS 2016, follow-up till 2022

1 parent/child

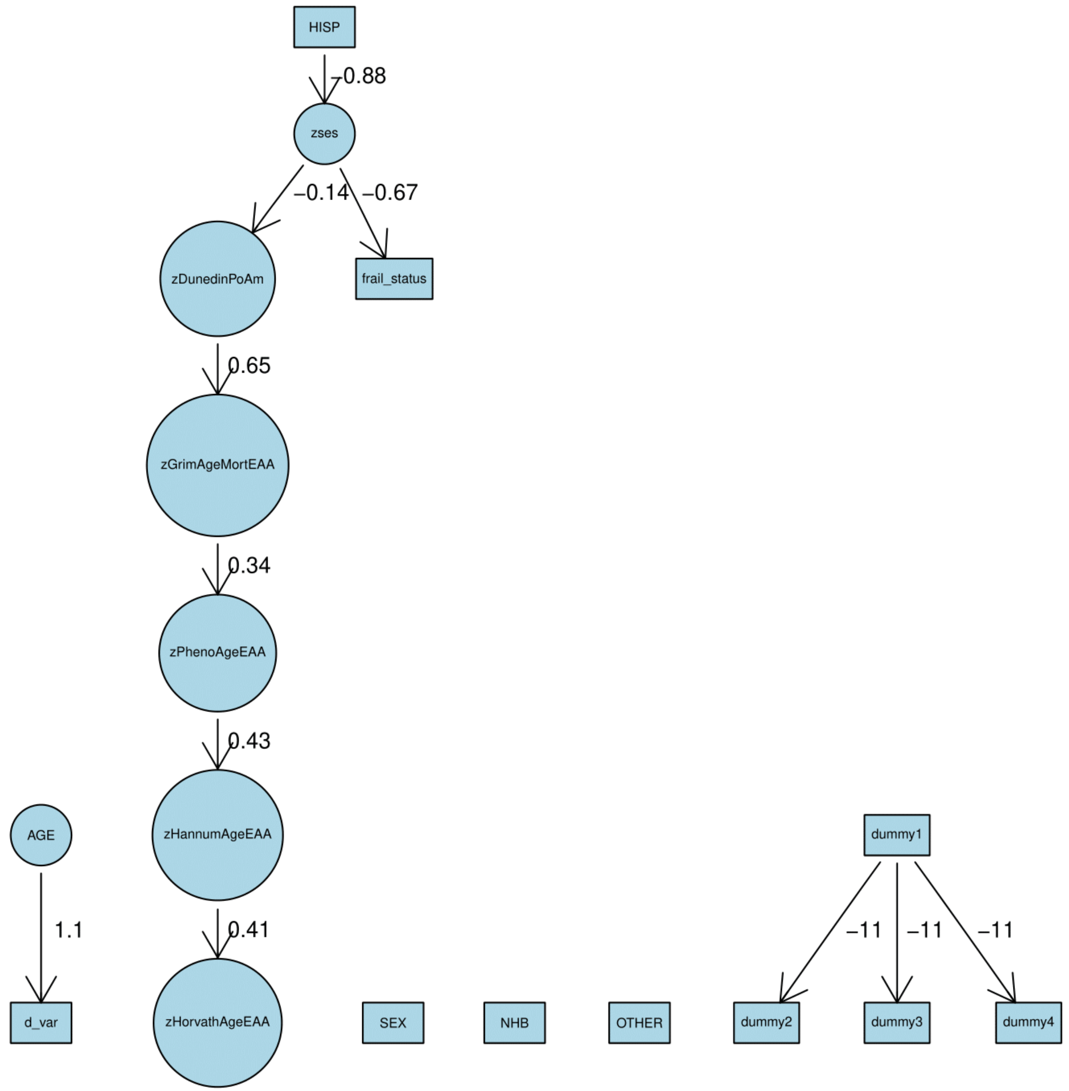

2 parents/child

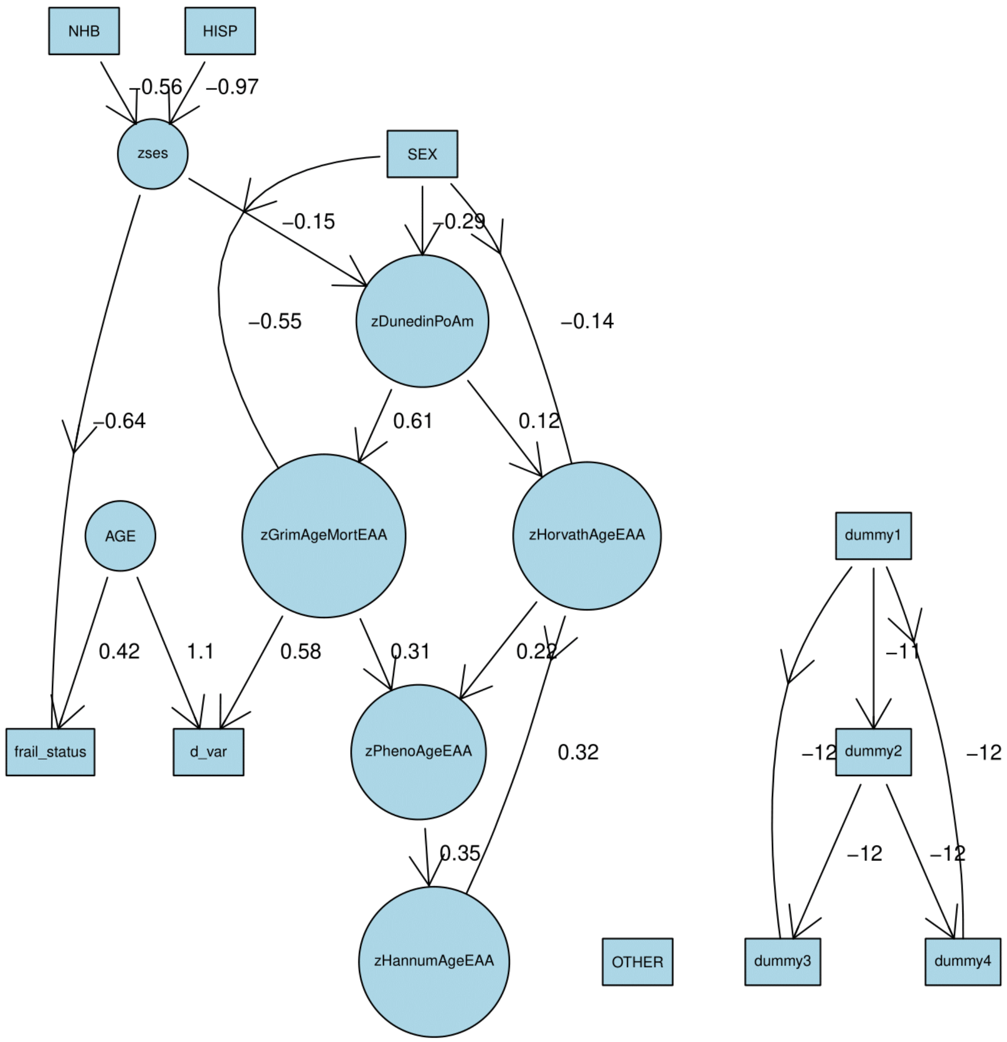

HRS 2016-2022: Final solution

3 parents/child solution

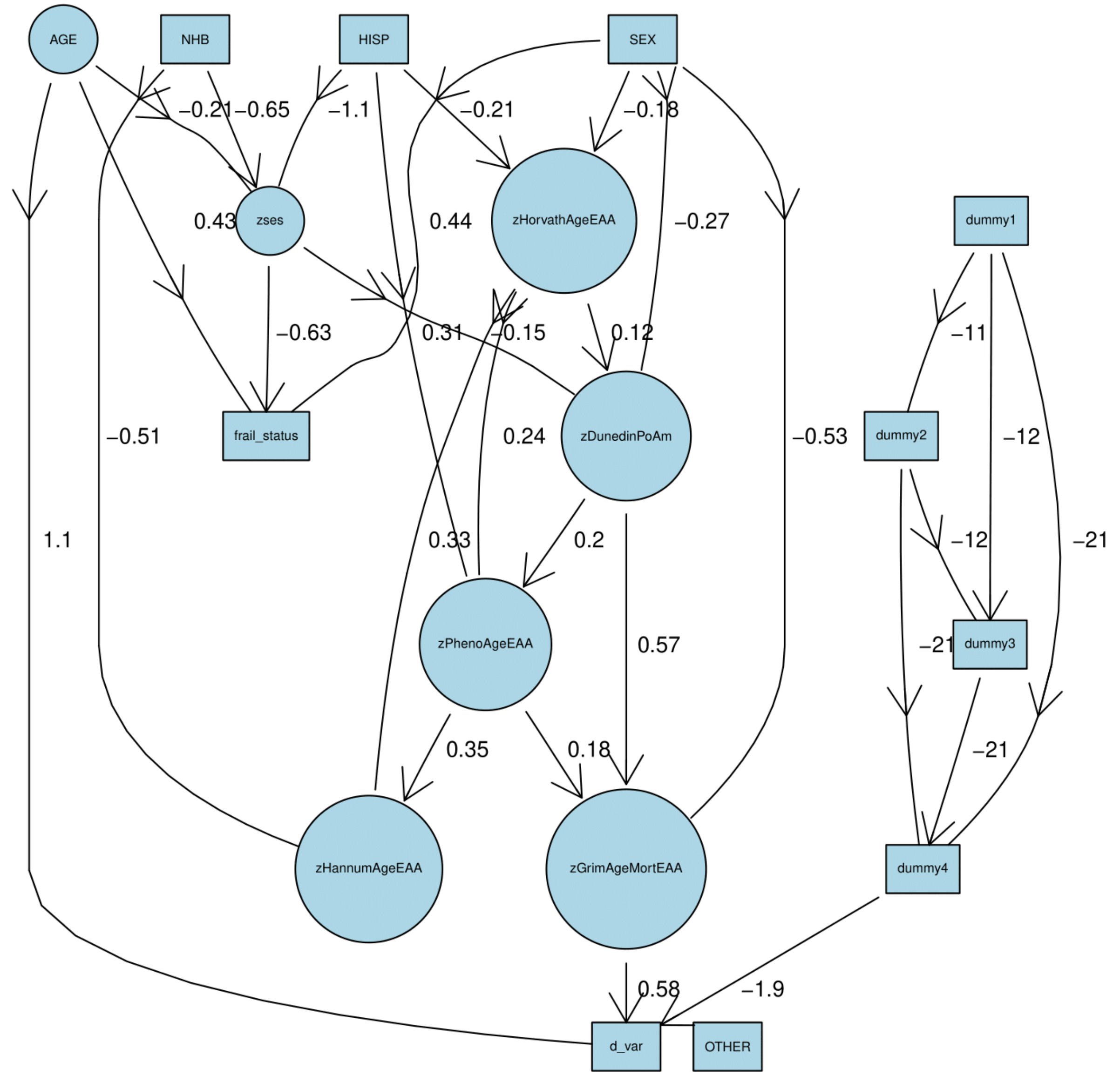

Model fit for 1-3 parents/child

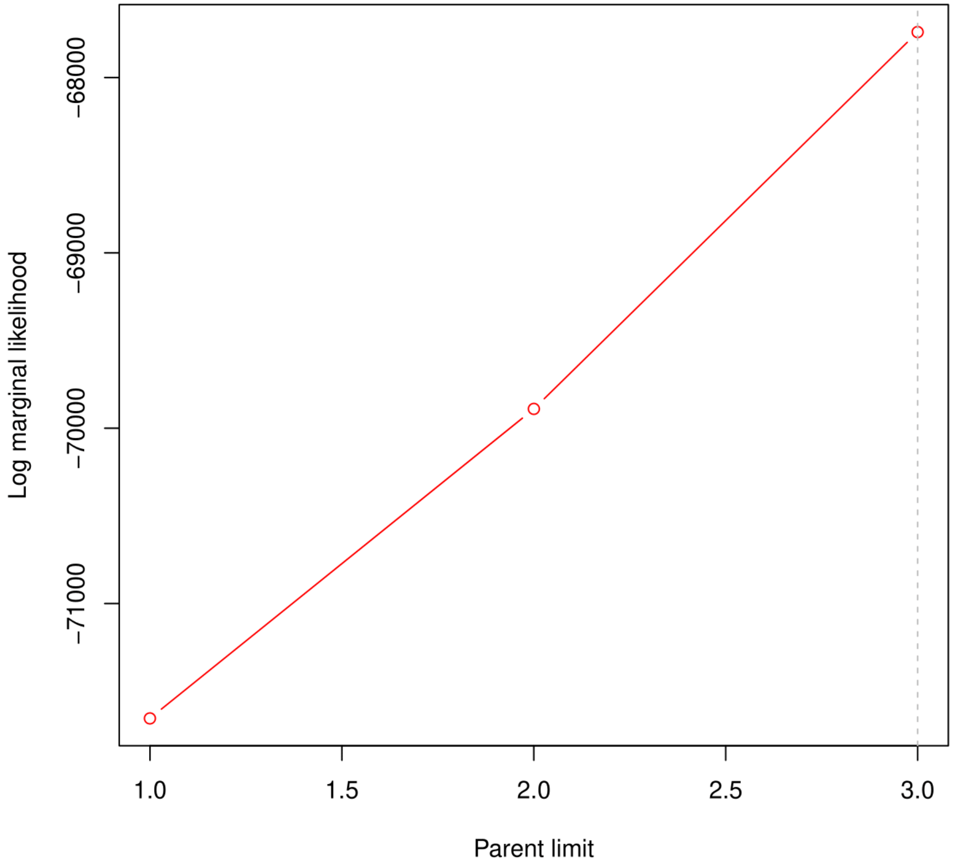

Notes: Details for R code used for this analysis described in **Appendix VI** and provided on Github. This code provides a comprehensive pipeline for conducting ABN analysis, including installation, data preprocessing, constraint specification, model fitting, and iterative optimization. It involves installing R versions 4.4 or higher, data preparation, data wrangling, defining variable groups, setting constraints, optimizing across parent limits, building the additive Bayesian network, and generating visual representations. The optimal number of parents of a child is determined based on levelling off the log marginal likelihood and desired complexity between key variables. Unweighted sample sizes were n=1,537 for NHANES, n=1,415 for HRS.

Abbreviations: DunedinPoAm=Dunedin Pace of Aging DNA methylation clock; frail_status: Frail=1, Pre-frail/Robust=0; GrimAgeEAA=Grim DNA methylation Epigenetic Age Acceleration; HannumAgeEAA=Hannum DNA methylation Age, Epigenetic Age Acceleration; HorvathAgeEAA=Horvath DNA methyalation Age, Epigenetic Age Acceleration; HRS=Health and Retirement Study; NHANES=National Health and Nutrition Examination Surveys; PhenoAgeEAA=Pheno DNA methylation Age Epigenetic Age Acceleration; SES=Socio-economic Status; z=standardized z-score.
