# Supplementary material for: Epigenetic aging markers in the association between frailty and mortality among U.S. adults
Source: BMC Med. 2026 Apr 15;24:323. doi: 10.1186/s12916-026-04866-0 (PMC13192009; doi:10.1186/s12916-026-04866-0)

Figure S4. Combined heatmaps of four-way decomposition models across primary and sensitivity analyses.

This figure presents four heatmaps summarizing four-way decomposition components for the frailty–epigenetic aging–mortality pathway across NHANES and HRS. Panels include: (A) primary analysis ; (B) primary analysis additionally adjusted for leukocyte composition (WBC) ; (C) reverse causation model (epigenetic aging → frailty → mortality) ; and (D) reverse causation model with additional WBC adjustment .

Rows represent epigenetic clocks (EPICLOCK), and columns represent four-way decomposition parameters, including the controlled direct effect (CDE), pure indirect effect (PIE), mediated interaction (INTmed), reference interaction (INTref), and total effect (TE), as well as corresponding proportion parameters (p_ and op_).

Color gradients indicate the magnitude and direction of standardized coefficients (blue = negative; red = positive for main effects; green–purple scale for proportion parameters). Values within cells denote coefficients rounded to three decimals; asterisks indicate statistical significance ($P < 0.05$).

Comparative visualization allows assessment of robustness to leukocyte adjustment and evaluation of directional consistency between primary and reverse causation frameworks.

(A) Primary analysis: Frailty → Epigenetic aging → Mortality

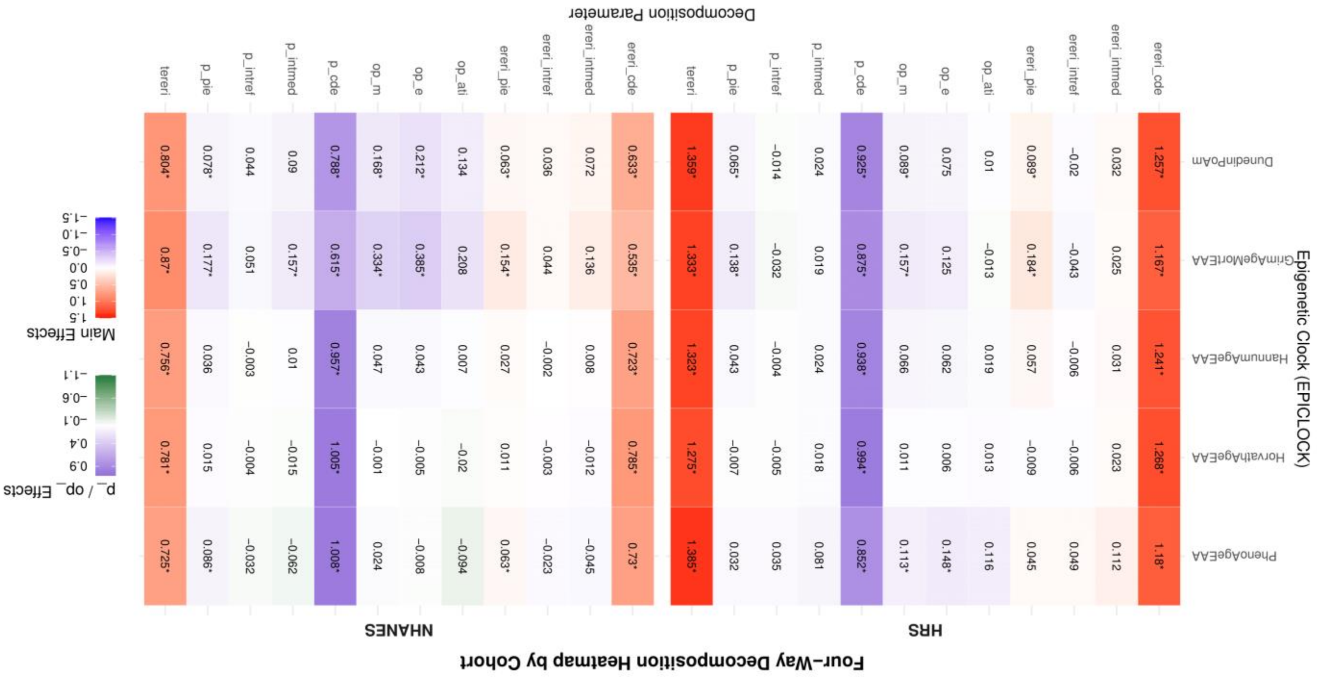

(B) Sensitivity analysis: Frailty → Epigenetic aging → Mortality: + WBC adjustment

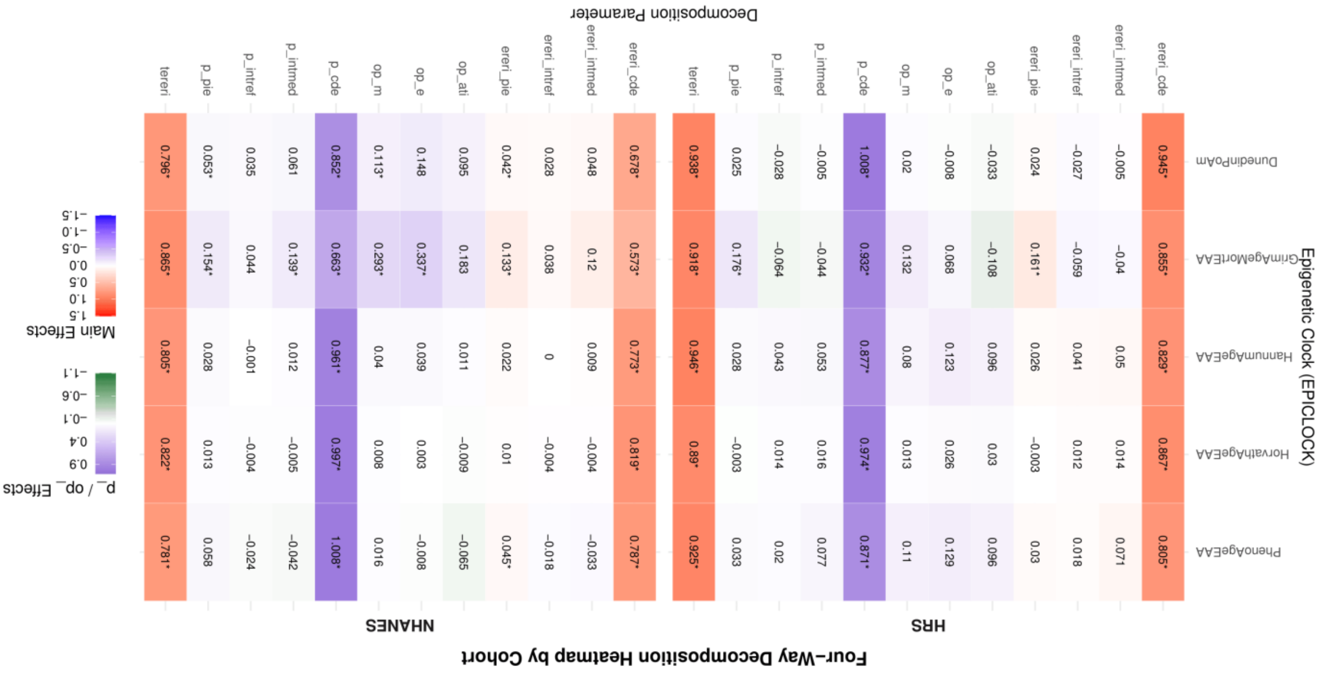

(C) Sensitivity analysis: Epigenetic aging →Frailty→ Mortality

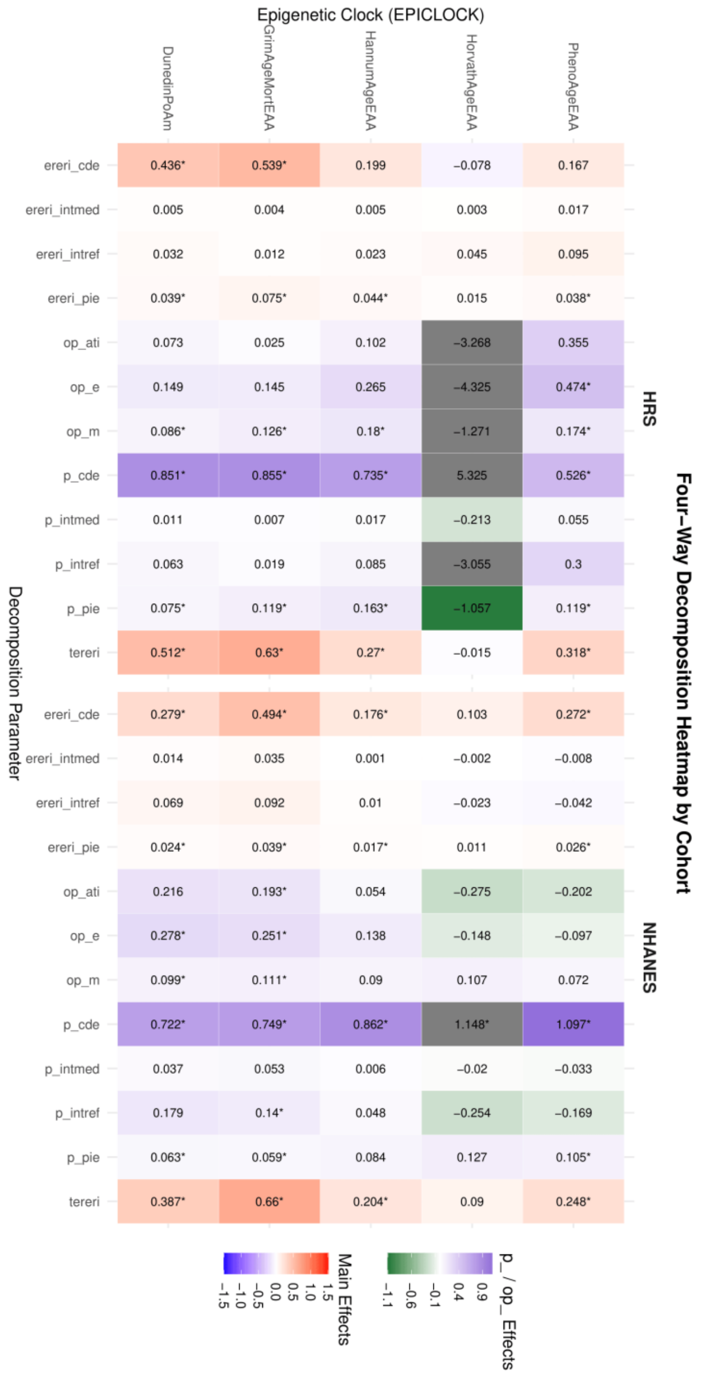

(D) Sensitivity analysis: Epigenetic aging →Frailty→ Mortality: + WBC adjustment

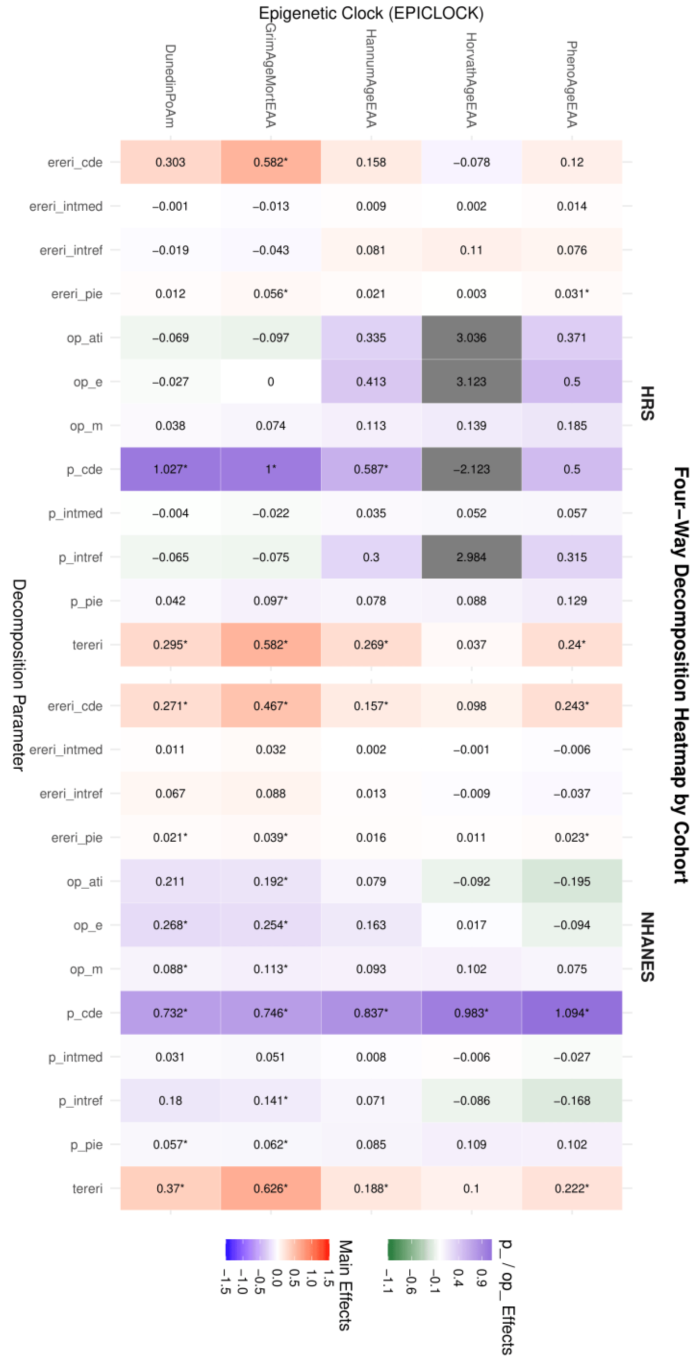

Supplement: Supplementary file 8 — Additional file 8: Figure S4. Fig. S4 – Heatmaps summarizing four-way decomposition results for frailty–epigenetic aging–mortality pathways across primary and sensitivity analyses, including leukocyte-adjusted and reverse-causation models. [file 12916_2026_4866_MOESM8_ESM.pdf]
